# Consensus approach to differential abundance analysis detects few differences in the oral microbiome of pregnant women due to pre-existing type 2 diabetes mellitus

**DOI:** 10.1099/mgen.0.001385

**Published:** 2025-04-15

**Authors:** Sophie M. Leech, Helen L. Barrett, Emily S. Dorey, Thomas Mullins, Josephine Laurie, Marloes Dekker Nitert

**Affiliations:** 1 School of Chemistry and Molecular Biosciences, The University of Queensland, St Lucia, QLD 4072, Australia; 2 Obstetric Medicine, Royal Hospital for Women, Randwick, NSW, Australia; 3 Faculty of Medicine, University of New South Wales, Sydney, NSW, Australia; 4 Mater Research Institute, The University of Queensland, South Brisbane, QLD, Australia; 5 Obstetric Medicine, Mater Health, South Brisbane, QLD, Australia

**Keywords:** diabetes mellitus, differential abundance analysis, oral microbiome

## Abstract

Oral microbiome dysbiosis has been proposed as a potential contributing factor to rising rates of diabetes in pregnancy, with oral health previously associated with an increased risk of numerous chronic diseases and complications in pregnancy, including gestational diabetes mellitus (GDM). However, whilst most studies examining the relationship between GDM and the oral microbiome identify significant differences, these differences are highly variable between studies. Additionally, no previous research has examined the oral microbiome of women with pre-existing type 2 diabetes mellitus (T2DM), which has greater risks of complications to both mother and baby. In this study, we compared the oral microbiome of 11 pregnant women with pre-existing T2DM with 28 pregnant normoglycaemic controls. We used shotgun metagenomic sequencing to examine buccal swab and saliva rinse samples at two time points between 26 and 38 weeks of gestation. To reduce variation caused by the choice of differential abundance analysis tool, we employed a consensus approach to identify differential taxa and pathways due to diabetes status. Differences were identified at the late time point only. In swab samples, there was increased *Flavobacteriaceae*, *Capnocytophaga*, *Capnocytophaga gingivalis* SGB2479, *Capnocytophaga leadbetteri* SGB2492 and *Neisseria elongata* SGB9447 abundance in T2DM as well as increased Shannon diversity and richness. In rinse samples, there was an increased abundance of *Haemophilus*, *Pasteurellaceae*, *Pasteurellales* and *Proteobacteria*. In contrast to studies of the oral microbiome in T2DM or GDM that use a single differential abundance analysis tool, our consensus approach identified few differences between pregnant women with and without T2DM.

## Data Summary

Raw sequence files with human DNA removed have been deposited in Sequence Read Archive with BioProject accession PRJNA1185841, which can be accessed at: https://www.ncbi.nlm.nih.gov/sra/PRJNA1185841. Scripts for differential abundance analysis as well as a brief metadata file containing diabetes status, body mass index and time point can be accessed at https://github.com/sophieleech/Differentially-abundant-taxa-for-T2DM-saliva.

**Impact Statement**

Previous investigations of the oral microbiome in gestational diabetes mellitus (GDM) have produced conflicting findings, which may be attributed to the choice of differential abundance analysis tool. In addition, no investigation of pre-existing type 2 diabetes mellitus (T2DM) in pregnancy has been conducted, despite the higher risk of pregnancy complications. In this work, we employ a consensus method to differential abundance analysis, which demonstrates that the choice of tool is a major source of variation between microbiome studies. Using this method, we identify relatively few differences associated with T2DM during pregnancy in the oral microbiome compared to normoglycaemic controls, in conflict with previous studies using single differential abundance analysis tools comparing normoglycaemic controls to women with GDM, a disease with overlapping pathophysiology. Whilst the number of differences was minimal, the use of a consensus method to differential abundance analysis makes our findings more robust than previously reported findings.

## Introduction

Like the gut microbiome, the oral microbiome is thought to play a significant role in human health. Not only is the oral microbiome important for oral health and the risk of gingivitis, periodontitis and caries, it is thought to also influence systemic health, with poor oral health being associated with the risk of developing certain cancers, cardiovascular disease, dementia and diabetes [1]. Additionally, poor oral health during pregnancy is associated with an increased risk of adverse pregnancy outcomes [2], and pregnant women are at an increased risk of poor oral health compared to non-pregnant women [3], with changes to the oral microbiota also reported [4,5]. Whilst previous findings vary, a meta-analysis reported that at least two studies found increases in taxa such as *Streptococcus mutans*, *Aggregatibacter actinomycetemcomitans*, *Porphyromonas gingivalis* and *Prevotella intermedia* in pregnant women compared to non-pregnant women, although across pregnancy, the oral microbiome composition appears to be stable in healthy women [6].

Several studies have previously investigated the link between gestational diabetes mellitus (GDM) and the oral microbiome. However, whilst most studies do identify significant differences, these differences are highly varied and often conflicting [7,11]. In our review of the literature, *Leptotrichia* was the most consistently identified and negatively associated with GDM in two studies [8,10], and an unidentified operational taxonomic unit of genus *Leptotrichia* was depleted in a third [7].

Like GDM, type 2 diabetes mellitus (T2DM) has also been associated with changes to the oral microbiota outside pregnancy, though also with highly varied findings between studies [12,18]. Rates of pre-existing type 2 diabetes in pregnancy have approximately doubled in western cohorts in the last two decades [19]. Whilst gestational diabetes and T2DM overlap in their pathophysiology, unlike GDM, pre-existing T2DM in pregnancy results in an entire pregnancy affected by hyperglycaemia. Due to this, women with pre-existing T2DM are at higher risk of experiencing complications during pregnancy such as large-for-gestational-age infants, pre-term delivery, pre-eclampsia, congenital malformations and neonatal hypoglycaemia [20,21]. However, to our knowledge, there are currently no studies reporting the composition and function of the microbiome in pregnant women with pre-existing T2DM.

Therefore, in the present study, we aimed to investigate the oral microbiome of women with pre-existing T2DM in comparison to normoglycaemic controls at two time points during pregnancy using two sampling methods. Two time points were included because the stability of the oral microbiome, as previously reported in healthy women across pregnancy, has not yet been examined in women with T2DM [6]. Both buccal swabs and saliva rinse samples were included, as saliva can somewhat capture multiple aspects of the oral microbiome due to its interaction with various niches [22,23]; structural changes to the buccal mucosa associated with T2DM motivate targeted analysis [24].

Additionally, numerous recent papers have highlighted the choice of differential abundance (DA) analysis tool as a major source of variation in results between studies [25,27]. As previous studies in this area have reported inconsistent findings, we speculate that the choice of DA analysis tool may play a role in this inconsistency. Therefore, to eliminate one source of inter-study variation, we will implement a consensus approach to DA analysis, reporting only differences identified by 50% or more of the DA analysis tools used. A comparison of DA tools was also conducted based on the number of consensus taxa detected to aid future researchers in the choice of a DA analysis tool or combination of tools.

## Methods

### Participant inclusion and sample collection

Women were recruited in their second trimester of pregnancy from the Mater Mothers’ Hospital and Royal Brisbane Women’s Hospital (Brisbane, Australia). The T2DM group consisted of women who were diagnosed with T2DM prior to their current pregnancy. The women in the control group consisted of normoglycaemic women without any previous diagnosis of diabetes mellitus including GDM, no history of abnormal glucose testing, no first-degree relatives with diabetes mellitus, no diagnosis of polycystic ovarian syndrome (PCOS) and hemoglobin A1c (HbA1c)<5.3% at screening. Participant characteristics are shown in Table 1. Following screening, participants attended three study visits for the collection of samples. The first occurred between 26 and 28 weeks of gestation (early time point), and the second occurred ~2 months later between 34 and 38 weeks of gestation (late time point). The final visit occurred within 1 week post-partum. In addition to clinical and demographic information, dietary intake information was collected and processed with the Cancer Council Victoria’s Dietary Questionnaire for Epidemiological Studies (V3.2) [28]. Fasting serum and plasma were also collected at both the early and late time points in pregnancy for measures of glucose and lipid metabolism. In total, there were 24 women with normoglycaemia and 9 women with T2DM with samples at both time points, 3 women with normoglycaemia and 2 women with T2DM with samples at the early time point only and a single normoglycaemic woman with samples at the late time point only.

**Table 1.** Characteristics of included women and their infants. The superscript number indicates alternate sample size due to data availability. Medical conditions include metabolic, endocrine, cardiovascular, inflammatory and mental health conditions. One patient receiving insulin had previously undergone a gastric bypass

	Control (*n*=28)	T2DM (*n*=11)	*P*-value
Age (years)	32 (29.3–35.8)	37 (31–39)	0.02
Pre-pregnancy BMI (kg m^−2^)	25.3 (22.9–28.3)	35.4 (27.9–38.9)	0.002
*BMI category*			
Underweight (BMI<18.5 kg m^−2^)	1 (3.6%)	0 (0%)	
Healthy weight (BMI 18.5<25.0 kg m^−2^)	12 (42.9%)	1 (9%)	
Overweight (BMI 25.0<30.0 kg m^−2^)	9 (32.1%)	2 (18.2%)	
Obese (BMI>30 kg m^−2^)	6 (21.4%)	8 (72.7%)	
Parity (previous pregnancy>20 weeks)	0 (0–1)	0 (0–2)	0.9
Gravidity (pregnancies ever, including current pregnancy)	2 (1–2)	3 (1–4)	0.1
Gestational age at delivery (days)	279.5 (272.3–287.8)	266.0 (262.0–268.0)	<0.0001
Caesarean section delivery (%)	10 (35.7)	8 (72.7)	0.07
Male infants (%)	14 (50)	6 (54.5)	>0.99
Infant birth weight (g)	3503 (3133–3830)	3560 (3205–3651)	0.94
Birth centile	42.0 (15.0–73.1)	64.6 (29.7–93.2)	0.1
*Diabetes management*			
Metformin only	–	1	
Insulin only	–	2	
Metformin and insulin	–	7	
Diet/lifestyle alone	–	1	
***Ethnicity***			0.06
Caucasian	24	5	–
Other	4	6	
Antibiotic use in pregnancy (%)	3 (10.7)	2 (18.2)	0.6
Other medical conditions (%)	11 (39.3)	8 (72.7)	0.08
**Diet**	***N*** **=21**	***N*** **=8**	
Energy (inc. fibre) (kJ day^−1^)	7997±1886	8070±2089	0.9
Carbohydrate (g day^−1^)	182.0±47.7	191.8±55.5	0.6
Protein (g day^−1^)	80.0±25.6	85.3±21.2	0.6
Fat (g day^−1^)	88.7±26.4	83.1±22.1	0.6
Dietary fibre (g day^−1^)	22.7 (16.6–29.2)	18.2 (16.1–20.8)	0.3
Smoker<3 months pre-pregnancy	1	2	0.2

	Early			Late		
	Control (***n*=27**)	T2DM (***n*=11**)	***P*-value**	Control (***n*=25**)	T2DM (***n*=9**)	***P*-value**
Gestational age at sampling (days)	196 (193–197)	191 (189–195)	0.07	253 (251–254.5)	251 (239.5–252.5)	0.04
HbA1c (%)	4.8 (4.7–4.9)^22^	6.2 (5.3–6.8)^10^	<0.0001	4.8 (4.7–4.9)^18^	5.6 (5.1–6.5)^6^	0.0002
Haemoglobin (mmol l^−1^)	117.6±6.0^26^	114.3±9.2	0.2	120.1±7.7^23^	114.4±12.2^8^	0.1
HDL (mmol l^−1^)	1.8 (1.6–2.2)	1.3 (1.0–1.8)^8^	0.006	1.8±0.3	1.7±0.3^6^	0.3
LDL (mmol l^−1^)	3.1±1.0	2.8±0.8^7^	0.4	3.4±0.8^23^	3.3±0.7^5^	0.8
Triglyceride (mmol l^−1^)	1.9 (1.5–2.3)	2.5 (2.1–2.9)^8^	0.02	2.4 (2.0–3.1)	3.2 (2.7–3.9)^6^	0.06
Total cholesterol (mmol l^−1^)	5.9±1.1	5.7±1.1^10^	0.8	6.4±0.98	6.4±1.2^8^	0.98
Fasting glucose (mmol l^−1^)	4.1 (4.0–4.4)	4.4 (4.1–5.5)^9^	0.1	4.2 (3.9–4.3)	4.5 (3.7–5.6)	0.3
Systolic BP (mmHg)	108.3±7.4	117.0±13.0	0.08	111.6±9.9	126.4±9.1^8^	0.0008
Diastolic BP (mmHg)	66.6±7.0	75.0±9.8	0.01	69.8±7.5	79.0±8.5^8^	0.006

In the event of missing data points, the actual sample size for comparison is represented as a superscript, e.g. 8 indicates *n*=8. If data is normally distributed, it is presented as mean±sd; otherwise, it is presented as median (interquartile range (IQR)). For comparison between two normally distributed groups, the *P*-value was calculated via unpaired t-test; otherwise, the *P*-value was calculated with the Mann–Whitney test. For categorical comparisons, the *P*-value was calculated by Fisher’s exact test or chi-square test.

### Collection of oral samples

Participants were asked to refrain from eating or drinking for 1 h prior to collection. Prior to collection, patients were instructed to rinse their mouth with bottled water. Buccal swab samples were allowed to dry inside the tube with a cap open for 10 min prior to freezing at −80 °C. For rinse samples, participants were instructed to gargle 10 ml of 0.9 w/v sterile saline for 1 min before expectorating into a sterile collection container. Samples were placed on ice before storing at −80 °C.

### Extraction of DNA from oral samples

DNA was extracted from oral swabs using the QIAamp DNA Mini Kit (QIAGEN, Hilden, Germany). Briefly, swabs were submerged in 400 µl of PBS. Twenty microlitres of proteinase K and 400 µl of buffer AL were added and vortexed for 15 s. Samples were incubated for 10 min at 56 °C. Four hundred microlitres of ethanol were added, and samples were vortexed and centrifuged. Samples were passed through a spin column, followed by 500 µl of buffer AW1 and buffer 500 µl of AW2. DNA was eluted in 50 µl of AE buffer and stored at −20 °C.

Saliva rinse samples were similarly processed: 2 ml of saliva was added to 3 ml of PBS and centrifuged for 5 min at 1800 ***g***. The supernatant was decanted, and the pellet was resuspended in 180 µl of PBS with 20 µl of proteinase K and 200 µl of AL buffer. The protocol then proceeded as for the saliva swabs. Extraction controls were included for all of the above. For oral swabs, the extraction was run with an unused sterile swab, whilst for the rinse control, extraction was run with PBS only.

### Microbiome analysis

Shotgun metagenomic sequencing was performed by the Centre for Microbiome Research (Brisbane, Australia). Sequencing was conducted with NovaSeq 6000 (Illumina, San Diego, California) using 2×150 bp paired-end chemistry to a target depth of 3 GB. Library preparation was performed with the Illumina DNA Prep kit (Illumina #20018705, San Diego, California). The actual returned depth was a median of 5.2 GB (IQR: 3.9–7.0 GB).

Raw sequences underwent quality control using ‘FastQC’ [29], ‘MultiQC’ [30], ‘Trimmomatic (0.39)’ [31], ‘Bowtie2 (2.4.5)’ [32], ‘Samtools view (1.6)’ [33] and ‘Samtools fastx (1.6)’ [33] to assess sequence quality, trim low-quality sequences, and remove adapter sequences and human contamination using default settings. MetaPhlAn4 (4.0.6) [34], with the database ‘mpa_vOct22_CHOCOPhlAnSGB_202212’, and HUMAnN 3.6 [35] were used to generate taxonomic and functional profiles with default settings. HUMAnN 3.6 was not able to generate pathway abundance for some normoglycaemic control samples, likely due to insufficient sequencing depth, and this reduces the sample size of pathway comparisons from 27 at the early time point to 26 in saliva rinse samples and 23 in buccal swab samples and from 25 at the late time point to 24 in swab samples. The R package ‘Decontam (1.24.0)’ [36] was used to identify contaminants for removal using the ‘combined’ method, with the default probability threshold of 0.1, which detects contaminants based on both frequency of taxa in samples with different DNA concentrations and prevalence of taxa in negative controls, where applicable profiles were then renormalized after removal of contaminants.

Following quality control, swab samples had an average of 375,199 (IQR: 253,956–597,345) paired reads, whilst rinse samples had an average of 643,574 (IQR: 409,849–1,489,253) paired reads. The majority of reads removed during quality control were sequences of human origin, which amounted to 97.79% (IQR: 96.91–98.09%) of swab sample reads and 96.38% (IQR: 94.52–97.63%) of rinse sample reads.

### Statistical analysis

#### Beta diversity

Beta diversity analysis was conducted using packages mixOmics (6.28.0) [37], phyloseq (1.48.0) [38] and vegan (2.6–6.1) [39]. For beta diversity analysis, functional profiles were analysed as reads per kilobase (RPK). Pseudo-counts of 0.0001% and 1 were applied to taxonomic and functional profiles, respectively, prior to centred log-ratio (CLR) transformation for beta diversity analysis. Statistical comparisons of beta diversity were made by generating Euclidean distance matrices of CLR values (Aitchison distances). For comparisons of matched and unmatched samples across time points, statistical analysis was conducted in GraphPad Prism 10 using either an unpaired t-test or a Mann–Whitney test. For group comparisons, ‘adonis2’ (vegan) with 9,999 permutations was used alongside ‘betadisper’ (vegan) to test for homogeneity of variances. Adonis2 results are reported only if the results of ‘betadisper’ were non-significant.

#### Alpha diversity

Due to the high sensitivity of richness to sequencing depth, with a strong correlation between sequencing depth and richness in both swab and rinse samples (Fig. S1 available in the online Supplementary Material), sequences were rarefied to the lowest post-quality control sequencing depth (81,879 reads) prior to alpha diversity analysis. Alpha diversity analysis was conducted on species genome bins (SGBs) as defined by MetaPhlAn4. Alpha diversity was calculated using the Shannon diversity index, richness and evenness in R, with GraphPad Prism 10 and R used for statistical analysis. Fitting of linear and linear mixed effect models was conducted in R using ‘lme4’ [40] and ‘lmerTest’ [41]. Comparisons of group characteristics were also conducted in GraphPad Prism 10. Data is presented as mean±sd if normally distributed or median (IQR) if non-normally distributed. Data was considered normal if it passed the Shapiro–Wilk test, the Kolmogorov–Smirnov test, the D’Agostino and Pearson test and the Anderson–Darling test.

#### DA analysis

In view of recent literature highlighting the high variation between DA analysis and the lack of a gold standard tool for differential analysis, we adopted a consensus approach for DA analysis [25]. DA analysis was conducted using ANCOM-BC2 (2.6.0) [42], MaAsLin2 (1.18.0) [43], ALDEx2 (1.36.0) [44,45], DESeq2 (1.44.0) [46], LinDA (MicrobiomeStat 1.2) [47] and ZicoSeq (GUniFrac 1.8) [26]. Taxonomy and pathways are reported in the main text only if they are identified as significant by 50% or more (≥3) tools, with results detected by only one or two of the algorithms reported in the supplementary results only. This threshold was chosen as it represents a simple majority and does not depend on the assumption that all tools are equally capable of detecting all true differences. For all tools, taxa were considered significantly differentially abundant if false discovery rate (FDR)-corrected *P*-values (q-values) were <0.05, with the default method of FDR used for each tool. Each taxonomy level was analysed separately. A comparison of the methods used by DA tools included in this study is outlined in Table 2.

**Table 2.** Comparison of DA analysis tools used in the present study

Tool (version)	Input	Method	Hypothesis test	FDR
MaAsLin2 (1.18.0)	Proportion or counts	Proportion: linear model of arcsine square root transformation (AST) data.Count: linear model of CLR-transformed data.	T-test with Satterthwaite’s degrees of freedom method (when random effects are implemented) or Wald’s test (without random effects)	Benjamini–Hochberg
ANCOM-BC2 (2.6.0)	Counts	Log-linear model accounting for sample-specific and taxa-specific bias.	Wald’s test	Holm–Bonferroni
ALDEx-2 (1.36.0)	Counts	Counts are converted to probabilities by Monte Carlo sampling from the Dirichlet distribution to account for sampling error. These probabilities are then CLR transformed. When covariates are included, a generalized linear model is then implemented.	Wilcoxon rank-sum or Kruskal–Wallis tests (aldex.glm)	Benjamini–Hochberg
DESeq2 (1.44.0)	Counts	Read counts are modelled as following a negative binomial distribution using logistic regression.	Wald’s test	Benjamini–Hochberg
LinDA (MicrobiomeStat 1.2)	Proportion or counts	Linear regression of CLR-transformed data with identification of bias term for compositional effect. When counts are used as input, zeros can be imputed based on library size.	Student’s t-test	Benjamini–Hochberg procedure
ZicoSeq (GUniFrac 1.8)	Proportion or counts	Uses taxa that are less likely to be differential as references to generate ratios with other taxa to perform DA analysis to account for compositionality. When the input is count data, an empirical Bayes model is used to estimate the underlying proportions. Linear model-based permutation test (Smith permutation).	F-statistic with permutation-based FDR control	

#### MaAsLin2 (1.18.0)

Relative abundance profiles from MetaPhlAn4 were first transformed to proportions (out of 1), and an AST was applied. A minimum prevalence threshold of 0.1 with a minimum abundance threshold of 1×10^−15^ was applied, as the prevalence threshold did not seem to apply correctly without an abundance threshold. *P*-values were adjusted using the Benjamini–Hochberg procedure. For comparison of time points and sample type, sample ID was included as a random effect. The effect of diabetes status was analysed with and without the inclusion of body mass index (BMI) as a fixed effect.

#### ANCOM-BC2 (2.6.0)

As ANCOM-BC2 requires counts and the output of MetaPhlAn4 is relative abundance, approximate counts were calculated by multiplying the number of sample sequences by relative abundance. RPK was used for pathways. A prevalence threshold of 0.1 was applied. As each group of interest was <50 participants, the pseudo-count filter was not applied in line with the recommendations by ANCOM-BC2 developers [42]. By default, the Holm–Bonferroni method was used to adjust *P*-values. For comparison of time points and sample type, sample ID was included as a random effect. The effect of diabetes status was analysed with and without the inclusion of BMI as a fixed effect.

#### ALDEx2 (1.36.0)

Approximate counts and RPK were used as in ANCOM-BC2, with the addition of rounding to meet the tool’s integer requirement. Prior to analysis, a 0.1 prevalence filter was applied. Approximate counts were CLR-transformed with ‘aldex.clr’ with ‘mc.samples = 1000’ and ‘gamma=0.25’. To determine differentially abundant taxa, ‘aldex.ttest’ and ‘aldex.effect’ were used. For comparison of time points and sample type, paired analysis was implemented. Benjamini–Hochberg-corrected *P*-values from the Wilcoxon rank-sum test were used to determine significance. For analysis of diabetes status with inclusion of BMI, ‘aldex.glm’ and ‘aldex.glm.effect’ functions were used.

#### DESeq2 (1.44.0)

Approximate counts and RPK were used as in ANCOM-BC2. Prior to analysis, a 0.1 prevalence filter was applied. Default settings were used, with ‘test=Wald’ and ‘sfType=poscounts’ to allow for analysis despite all taxa/pathways containing at least one zero value. *P*-values were corrected with the Benjamini–Hochberg procedure by default. As DESeq2 does not allow for random effects, sample ID was included as a fixed effect for time point and sample type analysis. The effect of diabetes status was analysed with and without the inclusion of BMI as a fixed effect.

#### LinDA (MicrobiomeStat 1.2)

As in MaAsLin2, profiles were first transformed to proportions (out of 1), and the ‘linda’ tool was used with ‘feature.dat.type = proportion’ and a prevalence filter of 0.1. By default, *P*-values were corrected with the Benjamini–Hochberg procedure. For comparison of time points and sample type, sample ID was included as a random effect. The effect of diabetes status was analysed with and without the inclusion of BMI as a fixed effect.

#### ZicoSeq (GUniFrac 1.8)

As in MaAsLin2, profiles were first transformed to proportions (out of 1). The ‘ZicoSeq’ tool was used with ‘feature.dat.type = ‘proportion’’ and ‘prev.filter = 0.1’. As outlined in the ZicoSeq vignette for proportional data, a square root transformation was applied [48]. All other parameters were left as default. For analysis of diabetes status with adjustment for BMI, ‘adj.name’ was set to BMI. For analysis of time point and sample type, ‘strata’ was set to sample ID.

#### Effect of input type

To determine the effect of the input format (approximate counts vs proportions), MaAsLin2, LinDA and ZicoSeq were also run with approximate counts and pathways as RPK as input with adjusted settings to allow for this. For count input, MaAsLin2 was run with a CLR transformation, LinDA was run with ‘feature.dat.type = count’ and ‘zero.handling = imputation’ and ZicoSeq was run with ‘feature.dat.type = count’; square root transformation was applied as in the ZicoSeq vignette for counts, and posterior sampling was enabled to account for differences in sequencing depth [48].

For brevity, the use of proportion data for MaAsLin2, LinDA and ZicoSeq will be referred to as method 1, whilst the use of count data will be referred to as method 2.

#### Effect of sequencing depth

As further discussed in the Results section, due to the significant difference in sequencing depth between normoglycaemic controls and T2DM swab samples (Fig. S2AB), swab analysis was also repeated with a subset of controls of greater sequencing depth to eliminate this difference. For swabs at the late time point, all controls with a sequencing depth greater than or equal to the lowest T2DM sample were included (*n*=13 controls and *n*=9 T2DM) (Fig. S2F). However, due to the larger difference at the early time point, only the top 10 samples with the greatest sequencing depth were selected from each group (Fig. S2E).

## Results

### Participant characteristics

Participant characteristics are shown in Table 1. Women with T2DM were significantly older (*P*=0.02) and had a higher pre-pregnancy BMI (*P*=0.002), HbA1c (early: *P*<0.0001; late: *P=*0.0002) and triglycerides at the early time point (*P*=0.02). Diastolic blood pressure was higher in women with T2DM at both the early (*P*=0.01) and late time points (*P*=0.006), systolic blood pressure at the late time point (*P=*0.0008) and an overall higher incidence of PCOS (*P*=0.02). Women with T2DM had lower high-density lipoprotein (HDL) at the early time point (*P*=0.006) and gestational age at delivery (*P<*0.0001). Samples from women with T2DM were also collected 2 days earlier on average than control samples at the late time point (*P=*0.04), though this difference is unlikely to be clinically meaningful.

### Sample type

**Fig. 1.**
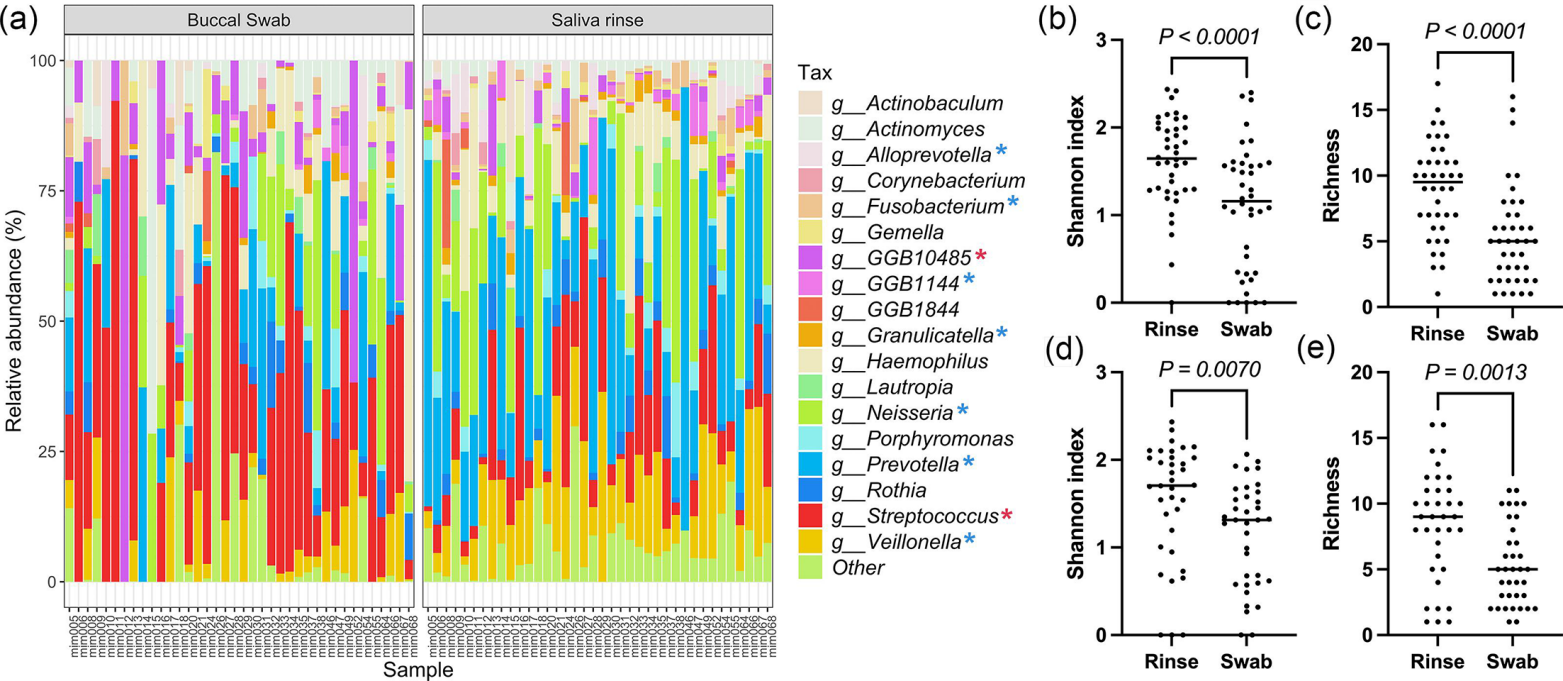
(a) Microbiome of swab and rinse samples at the early time point at the genus level. Other represents taxa present in <5% samples and/or present at <3% abundance. The red asterisk (*) represents a significant increase in swab, and the blue asterisk (*) represents an increase in rinse. Alpha diversity of rarefied rinse samples compared to rarefied swab samples at early (b, c) and late (d, e) time points measured by (b, d) the Shannon index and (d, e) richness. *P*-values were calculated by the Wilcoxon test. The horizontal bar represents the median.

Oral swab and rinse samples captured different niches of the oral microbiome with visible differences in composition (Fig. 1a). Following rarefaction, rinse samples had significantly greater Shannon index at both time points compared to swab samples (early: *P*<0.0001; late: *P*=0.007, Fig. 1b, d) and richness (early: *P*<0.0001; late: *P*=0.0013, Fig. 1c, e). A number of taxa and functional pathways were found to be significantly differentially abundant between sample types at both time points (Tables S8–S35).

### Time point

There was minimal change in the oral microbiome between early and late time points, with no difference in alpha or beta diversity of taxonomy or functional pathways, in swab or rinse samples across time points when analysing the whole cohort or T2DM and control samples independently. Additionally, there were no differentially abundant consensus taxa or pathways across time points, with no significantly different taxa or pathways identified in ≥50% of tools. Analysing T2DM and control samples separately also did not produce any time point differences.

**Fig. 2.**
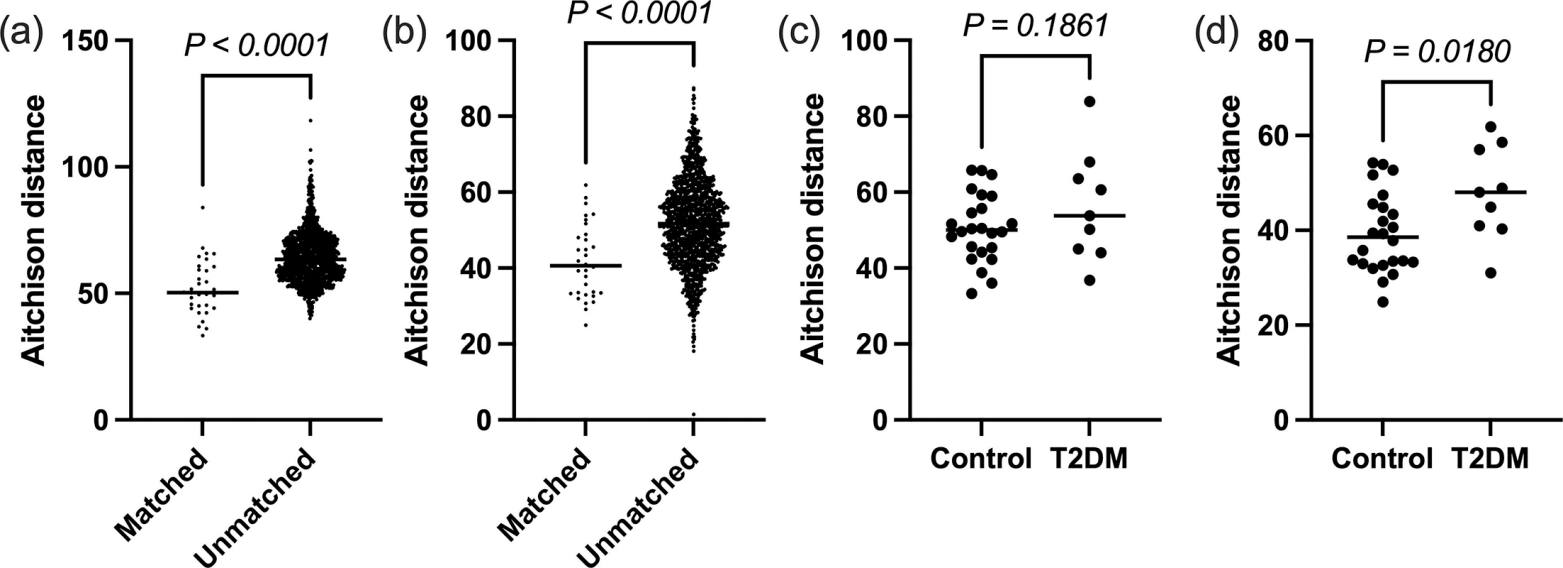
Aitchison distances from taxonomic profiles between matched and unmatched oral microbiome CLR-transformed data across time points in (a) rinse and (b) swab samples. Aitchison distances between matched samples across time points in normoglycaemic controls and T2DM women in (c) rinse and (d) swab. *P*-values were calculated by (a) the Mann–Whitney test and (b–d) the unpaired t-test. The horizontal bar represents the median.

Taxonomic stability across time points is also demonstrated by greater similarity of matched samples compared to unmatched samples across time points by Aitchison distance (*P*<0.0001) (Fig. 2a, b). This was also observed to a lesser degree when comparing Aitchison distances based on functional profiles of matched and unmatched rinse samples (*P*=0.036, Fig. S7A), but not for swabs (*P*=0.11, Fig. S7B).

Comparing the taxonomic Aitchison distance of the matched samples of T2DM to normoglycaemic controls across time points produced no difference in saliva rinse samples (Fig. 2c) but demonstrated that the buccal microbiome of controls was more stable than T2DM (Fig. 2d) between time points (*P=*0.018). Matched Aitchison distances from functional profiles did not differ, however, in either rinse or swab samples when comparing T2DM to controls (Fig. S7AD).

### Diabetes status

**Fig. 3.**
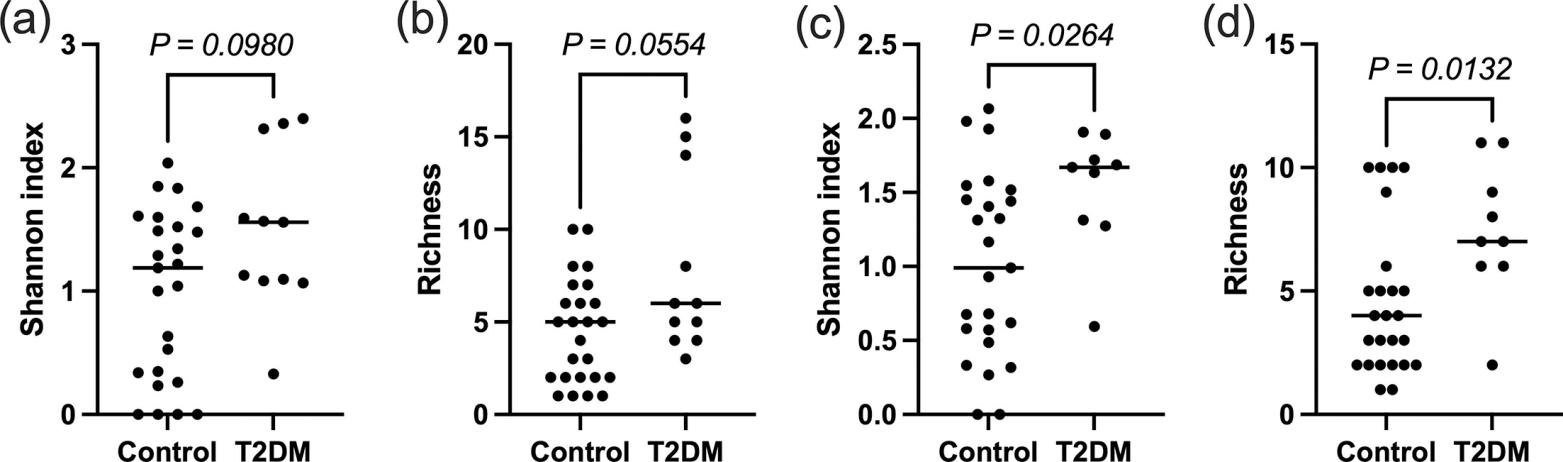
Shannon index and richness of rarefied swab samples of normoglycaemic and type 2 diabetic mothers’ buccal microbiome at (a, b) early and (c, d) late time point in the whole cohort. *P*-values were calculated by the Mann–Whitney test. Horizontal bars represent the median.

Diabetes status had minimal impact on saliva diversity, with no difference in alpha or beta diversity of taxonomy or functional pathways in rinse samples at either time point. There were no differences in beta diversity of taxonomy or functional pathways in swab samples; however, there was an increase in richness and the Shannon diversity index at the late time point in T2DM swab samples compared to normoglycaemic controls (Fig. 3c, d). Notably, at the early time point, richness was also increased in T2DM compared to controls with corresponding increases in the Shannon index; however, this was borderline non-significant (*P*=0.055, Fig. 3a, b).

**Fig. 4.**
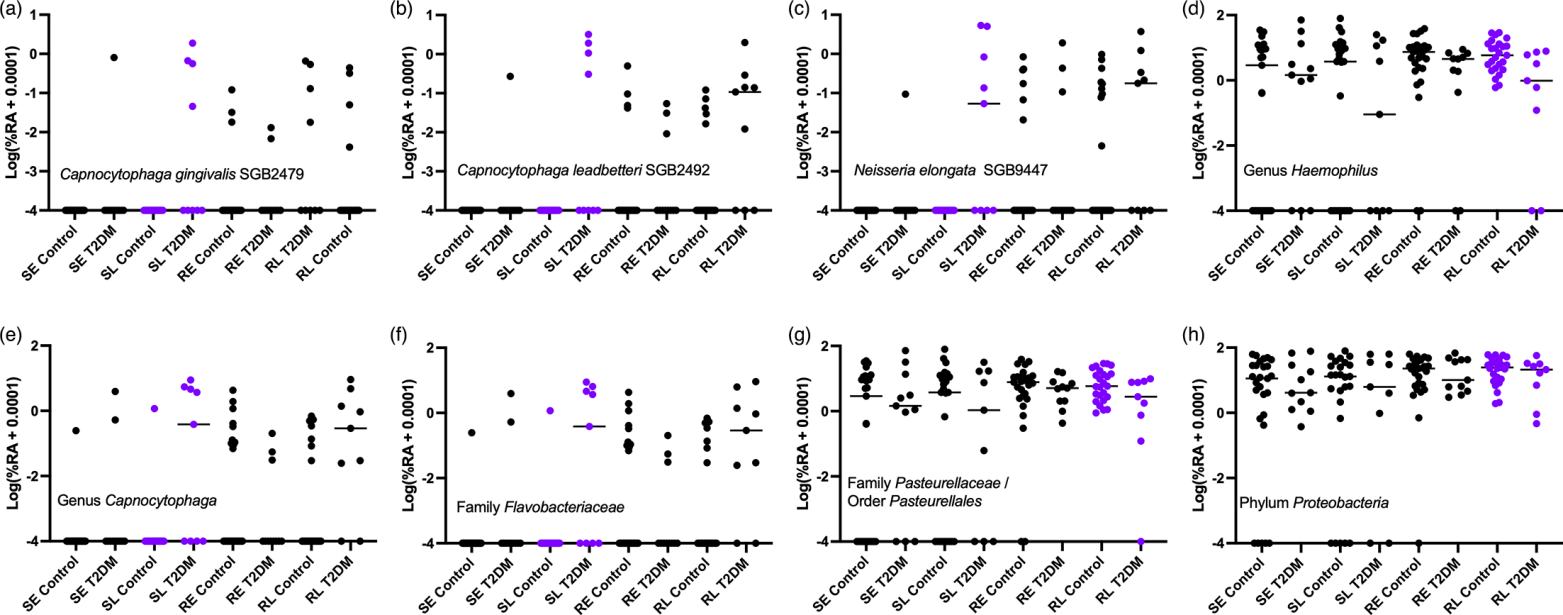
Log(relative abundance (%)+0.0001) of significantly differentially abundant taxa by diabetes status identified by ≥3 DA tools using method 1 or 2 across all sample types and time points. Significant comparisons are coloured purple, while all others are non-significant. Family *Pasteurellaceae* and order *Pasteurellales* are combined due to *Pasteurellales* being entirely made up of *Pasteurellaceae* in this cohort. The horizontal bar represents the median. S, swab; R, rinse; E, early time point; L, late time point.

Consensus differentially abundant taxa were identified at the late time point only. Using method 1, consensus differentially abundant taxa were identified in swab samples at the late time point, with consensus between three or four tools (MaAsLin2, ZicoSeq, LinDA and DESeq2). *Capnocytophaga*, *Capnocytophaga gingivalis* SGB2479, *Capnocytophaga leadbetteri* SGB2492 and *Neisseria elongata* SGB9447 were increased in T2DM (Fig. 4a–c, e). These were also identified as consensus taxa when count input was used for MaAsLin2, ZicoSeq and LinDA (method 2).

Using count data as input for all tools (method 2) resulted in additional taxonomy differences that were not consensus in method 1. In rinse samples at the late time point, ALDEx2, MaAsLin2 and LinDA identified *Haemophilus*, *Pasteurellaceae* and *Proteobacteria* as increased in controls, and these tools plus DESeq2 identified *Pasteurellales* as increased (Fig. 4d, g, h). *Flavobacteriaceae* was found to be increased in T2DM in swab samples at the late time point (Fig. 4f). In this study, *Flavobacteriaceae* was largely composed of the genus *Capnocytophaga*.

Notably, when present, all differentially abundant species were low abundance with a median of 0.7% (IQR: 0.3–1.3%), 1.1% (IQR: 0.29–2.5%) and 0.5% (IQR: 0.08–5.1%), respectively (Fig. 4a–c), and do not appear in the rarefied data used for alpha diversity analysis. This is troubling as there was a significant difference in the sequencing depth between controls and T2DM in swab samples with T2DM samples having a greater sequencing depth (Fig. S2AB). This was not the case for rinse samples, which had no difference in sequencing depth (Fig. S2CD). This raises the question of whether this difference is simply a product of the difference in sequencing depth resulting in non-detection in the control group. Rarefaction, whilst recommended prior to alpha diversity analysis, is not generally recommended prior to DA analysis, due to the loss of data [49], though it may be necessary when the sequencing depth correlates with the grouping of interest [26]. As described above, the species of interest do not appear following rarefaction to the level used in the alpha diversity analysis. Hence, to examine this relationship, we would need to select a higher threshold, resulting in the loss of both sequencing depth and samples, both contributing to an overall loss of sensitivity. Instead, we took a subset of controls with sequencing depth comparable to the T2DM samples, as described in the Methods section, and repeated the analysis on this subset, reducing sample size but not sequence depth (Fig. S2EF). No significant differences were now detected between these two groups (Table S3), suggesting that the detected differences in the full cohort may indeed be a product of sequencing depth. However, whilst not significant, the rinse samples at the late time point follow a similar trend of increased *C. leadbetteri* SGB2492, *N. elongata* SGB9447, *Capnocytophaga* and *Flavobacteriaceae* in T2DM participants (Fig. 4), with no significant difference in sequencing depth across rinse samples (Fig. S2CD). Additionally, these taxa largely do not appear at the early time point despite similar sequencing depths across time points (Fig. S3D). Ultimately, this result needs to be confirmed in a cohort of greater sample size and depth.

For functional pathways, no differences were identified in all combinations of sample type and time point in ≥3 tools using either method 1 or 2.

Following adjustment for BMI, the only difference that remained was that *Flavobacteriaceae* increased in T2DM participants at the late time point in swab samples using method 2.

### Performance of DA tools

**Fig. 5.**
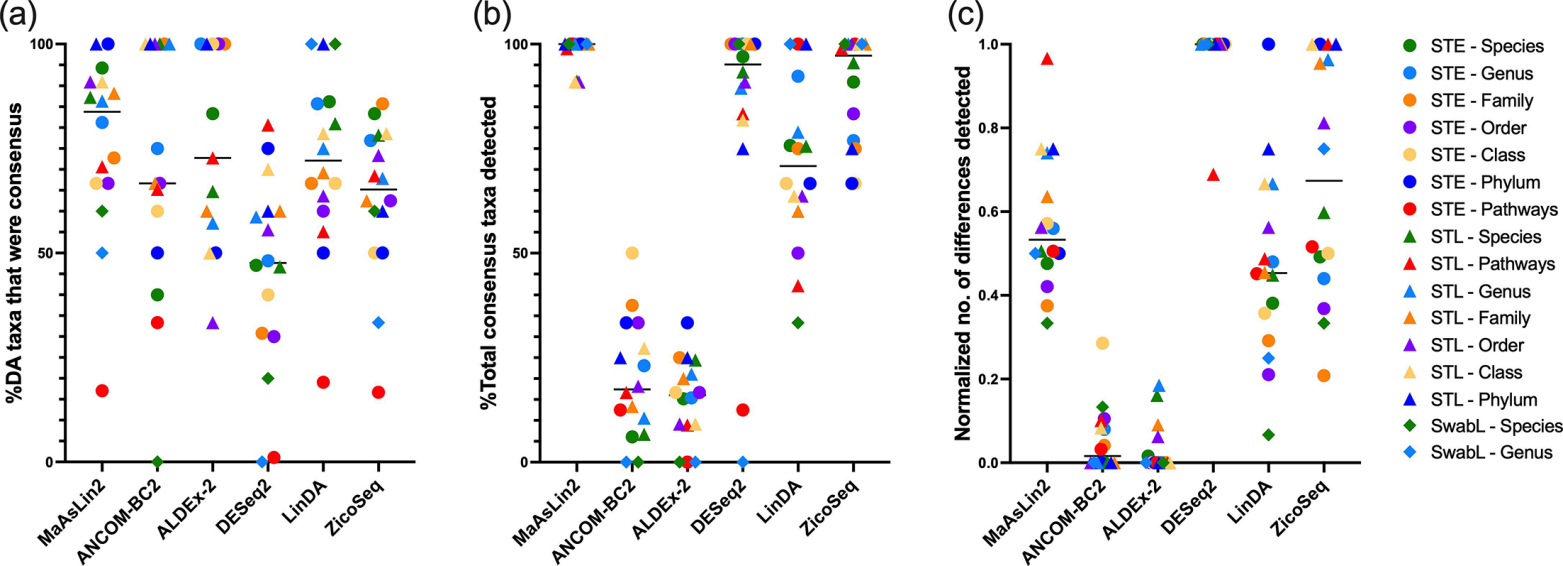
Comparison of DA analysis tools in the identification of consensus taxa/pathways when proportion data is used for MaAsLin2, LinDA and ZicoSeq (method 1). Consensus taxa/pathways are defined as those that were identified as differentially abundant by 50% or more tools. (a) Percentage of differentially abundant taxa/pathways detected by that tool that were consensus taxa/pathways. (b) Percentage of total consensus taxa/pathways identified by the tool. (c) Number of differences identified by the tool normalized so that the dataset with the maximum value=1 and the minimum value=0. STE/L: sample type at early/late time point; SwabL: diabetes status in swabs at late time point. Only datasets in which there were consensus taxa/pathways are included. The horizontal bar represents median.

Of the differential taxa identified by each tool using method 1, MaAsLin2 identified the highest proportion of consensus taxa with 83.8% (66.7–90.9%) of taxa identified being consensus taxa (Fig. 5a). Additionally, MaAsLin2 almost always identified all consensus taxa, on average identifying 100% (99.2–100%) of taxa (Fig. 5b). DESeq2 and ZicoSeq also identified almost all consensus taxa with 95.2% (82.2–100%) and 97.2% (75.5–100%) of consensus taxa identified on average, respectively (Fig. 5b). Whilst DESeq2 had a high identification of consensus taxa, it also identified many other taxa (Fig. 5c), which were not found by other tools (Tables S1–S7) resulting in the lowest average percentage of taxa being identified as consensus [47.6% (30.2–60.0%)] (Fig. 5a). This likely indicates a high FDR. ALDEx-2 and ANCOM-BC2 consistently identified the lowest number of taxa/pathways as differentially abundant (Fig. 4c) and therefore also tended to identify the lowest number of consensus taxa (Fig. 5b). Tools were most inconsistent when analysing pathway differences between sample types at the early time point (Fig. 5a), with less than 34% of pathways identified by each tool being consensus. Interestingly, there were instances where multiple tools would identify a taxon as significantly differentially abundant; however, the direction of change was inconsistent between methods.

**Fig. 6.**
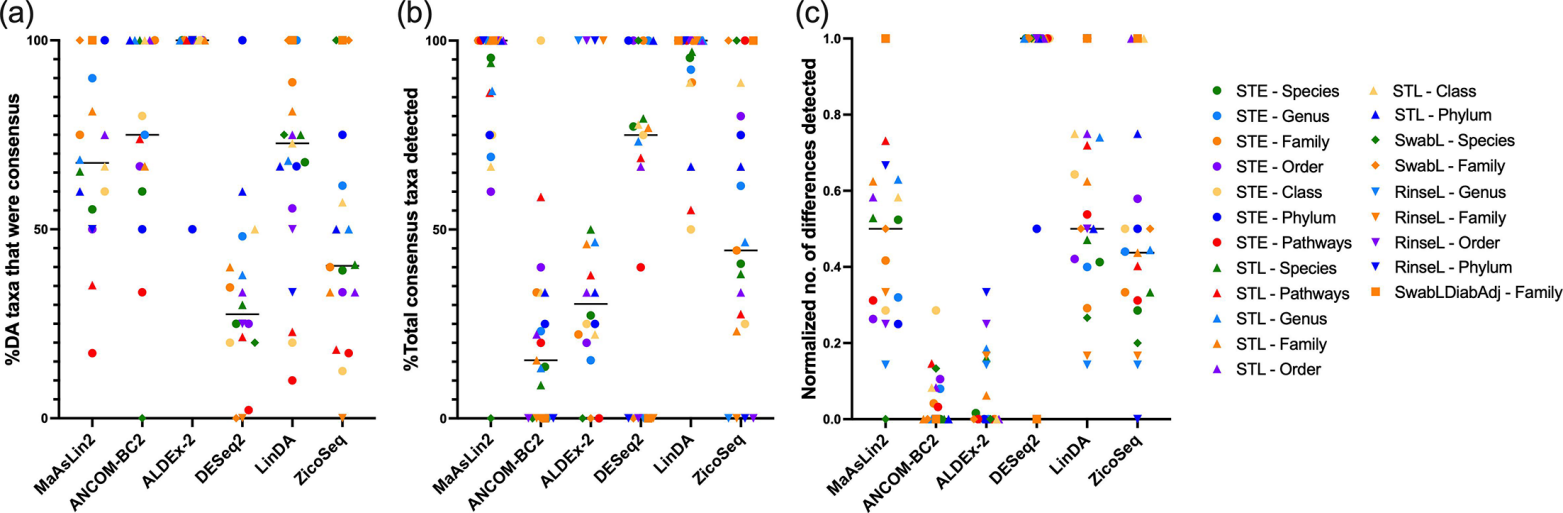
Comparison of differential abundance analysis tools in the identification of consensus taxa/pathways when all tools use count as input (method 2). Consensus taxa/pathways are defined as those that were identified as differentially abundant by ≥50% of tools. (a) Percentage of differentially abundant taxa/pathways detected by the tool that were consensus taxa/pathways. (b) Percentage of total consensus taxa/pathways identified by the tool. (c) Number of differences identified by the tool normalized so that the maximum value=1 and the minimum value=0 for each dataset. STE/L: sample type at early/late time point; SwabL: diabetes status in swabs at late time point. Only datasets in which there were consensus taxa/pathways are included. The horizontal bar represents the median.

As there was a trend towards agreement between MaAsLin2, LinDA and ZicoSeq, which all used proportions as input in this analysis, whilst other tools used approximate counts, the analysis was repeated with approximate counts as input for all tools (method 2) (Fig. 6), which also alters the methods used by LinDA, ZicoSeq and MaAsLin2 in the present study. This altered the perceived performance of some tools (Fig. 6), with ALDEx-2 having the highest proportion of differentially abundant taxa that were consensus, with 100% of differentially abundant taxa identified as consensus in all but one comparison (Fig. 6a). ZicoSeq and DESeq2 had the lowest agreement with other tools, with 40.3% (29.5–64.9%) and 27.5% (20.0–42.0%), respectively (Fig. 6a), of DA taxa identified as consensus. ZicoSeq’s perceived performance as measured by the percentage of consensus taxa identified also changed the most dramatically, with now only 44.4% (24.0–84.4%) of consensus taxa identified (Fig. 6b). In terms of total DA taxa identified (Fig. 6c), DESeq2 persisted as the highest, and ANCOM-BC2 and ALDEx2 the lowest.

Generally, most taxa that were identified as consensus with method 1 were also identified as consensus with method 2. This was particularly true for consensus taxa identified by ≥4 tools (Tables S8–S35).

## Discussion

To our knowledge, this was the first study examining the oral microbiome of women with pre-existing T2DM in pregnancy. Overall, our study identified a small number of differences associated with T2DM, but no significant changes in diversity measures, taxa or functional pathways across time points. Despite a lack of clear time point differences, differences associated with diabetes status were only identified at the late time point. In swab samples, women with T2DM had increased overall richness and increased abundance of *Flavobacteriaceae*, *Capnocytophaga*, *C. gingivalis* SGB2479, *C. leadbetteri* SGB2492 and *N. elongata* SGB9447, though these may reflect differences in sequencing depth compared to controls. Additionally, T2DM swab samples were less similar across time points compared to controls using Aitchison distances. In rinse samples at the late time point, there were significant depletions of *Proteobacteria*, *Pasteurellales*, *Pasteurellaceae* and *Haemophilus* (of family *Pasterellaceae* and order *Pasteurellales*) in T2DM compared to controls. Following adjustment for BMI, *Flavobacteriaceae* was the only remaining significant difference, though notably, in this cohort, this family is almost entirely composed of *Capnocytophaga*.

Previous studies have found no change in the Shannon diversity of saliva across pregnancy and minimal changes in taxonomy, aligning with our findings in the present study [4,50, 51]. We observe no difference in alpha diversity between T2DM and control in saliva rinse samples, similar to what has been reported in women with GDM [7,8]. However, in non-pregnant adults with T2DM and periodontal disease, T2DM was associated with decreased alpha diversity in saliva compared to controls in some [12,15], but not other [16], studies. As a significant decrease in alpha diversity of saliva associated with T2DM is predominantly identified outside pregnancy, but no differences are seen in pregnancy, this may suggest that pregnancy drives this lack of difference. Additionally, women with and without GDM were found to have greater Shannon diversity and richness in saliva than they did post-partum [7]; hence, this overall increase in alpha diversity in pregnancy may mask the decrease that occurs in association with T2DM outside of pregnancy. It is also possible that the presence of periodontal disease, which was not recorded in this cohort, is a driver for the differences observed outside pregnancy.

Although we observed no change in the alpha diversity of saliva rinse samples, we observed increased richness in buccal swabs of T2DM women compared to normoglycaemic controls at the late time point. Increased richness of both buccal and tongue dorsum swabs of non-pregnant T2DM individuals has also been reported [52,53]. Whilst not directly comparable to the changes that we see in the buccal mucosa, the tongue and buccal surface together comprise the oral mucosa and thus are more similar than samples from saliva or gingiva. Structural changes to the buccal and tongue mucosa due to T2DM have also previously been reported [24], and there is some evidence suggesting increases in oral mucosal lesions associated with diabetes [54]. These changes to the oral mucosa may drive the differences in buccal swab samples observed in the present study. Unfortunately, we were not able to identify other studies examining the buccal mucosa in GDM, so a comparison in pregnancy was not possible. The discrepancy between saliva rinse and buccal swab sample observation highlights the importance of recognizing the existence of multiple niches within the oral microbiome and investigating these independently.

*Capnocytophaga*, which was increased in the buccal swab samples from T2DM participants in the present study, has previously been reported to be increased in GDM [9,10]. Whilst this was in supragingival plaque rather than buccal swabs, it is likely that some supragingival plaque was also collected during buccal swabbing in the present study, given the close proximity of the two oral locations. In non-pregnant people with T2DM, one study has previously reported increases in *Capnocytophaga* in subgingival plaque and in saliva [17,55]. *Capnocytophaga* species have been linked to the development of both periodontal disease and oral cancer [56]. Specifically, *C. gingivialis*, which was increased in T2DM women in the present study, has been shown to not only be more abundant in oral squamous cell carcinoma (OSCC) but also to increase both invasion and migration of OSCC cells [56]. *Capnocytophaga* may be increased in association with periodontal disease and T2DM due to increases in available iron due to gum bleeding, which is required by *Capnocytophaga*, as well as increased glucose availability, which is the main facilitator of *Capnocytophaga* growth [57].

Whilst we could not find previous instances of an increase in *N. elongata* in association with T2DM, the genus *Neisseria* has been previously found to be increased in both buccal swabs and subgingival plaque [17,52]. However, *Neisseria* has also previously been reported to be depleted in tongue dorsum swabs from non-pregnant T2DM participants [58]. *Haemophilus* was decreased in saliva rinse samples of T2DM in the present study. *Haemophilus* has previously been found to be depleted in both saliva and tongue dorsum in non-pregnant T2DM participants [58], as well as increased in healthy controls compared to those with periodontal disease [55,59]. Phylum *Proteobacteria* was also decreased in saliva rinse samples, aligning with previous findings in non-pregnant T2DM individuals [60,61], and has also been found to be increased in mild periodontal disease compared to severe periodontal disease [59]. Whilst not evidence of a causal link, the decline of these bacteria in both T2DM and in periodontal disease is of interest, and further research is needed to elucidate the potential causal relationship between oral taxa and systemic health.

Overall, we identified fewer differences in the oral microbiome of women with pre-existing T2DM compared to normoglycaemic controls than previously identified when comparing pregnant women with GDM to normoglycaemic controls, or non-pregnant individuals with T2DM to normoglycaemic controls in studies using a single DA tool. Whilst this may be explained by our small sample size and low sequencing depth reducing our ability to detect differences in low-abundance taxa, the use of the consensus approach gives more confidence to our findings.

### Effect of DA methods on findings and considerations for future research

The results of DA analysis varied widely between tools, highlighting this as a major source of variation between published microbiome studies. However, there are a number of factors that need to be considered by future researchers.

The choice of consensus threshold will clearly influence the study’s findings, both in determining significant taxa and also in evaluating the performance of the tools as this was based on the ability to detect these arbitrarily defined consensus taxa. Simulation studies with pre-defined ‘true’ DA taxa would be beneficial to further support a consensus threshold, ideally on datasets that have been simulated in a variety of ways or using the recently proposed signal implantation simulation method, to combat previous critiques where the method of simulation influenced perceived tool performance [27].

One obvious criticism of using a consensus method for DA analysis is that just because multiple tools identify a taxon as differentially abundant, this does not indicate that it is a true positive finding, and the tools may just be picking up the same noise. However, as each tool operates with slightly different assumptions and strategies for overcoming the sparse, compositional nature of microbiome data, this seems unlikely. Additionally, as there is currently no agreed-upon gold standard for DA analysis, with varied reports of the performance of each tool depending on the dataset, the consensus method appears more conservative than the use of any single tool.

Whilst it may be tempting due to its ability to produce significant differences, we highly recommend against the solo use of DESeq2, which consistently identified far more significant differences than any other tool, even in situations where no other tool detected any differences, suggesting that it may have a high FDR. As a high proportion of previous studies in this area have utilized DESeq2 or LEFse, which has previously been demonstrated to be inconsistent across datasets and by default does not correct *P*-values to limit false discovery [25], this choice of DA tool may explain some of the variation in previous study findings [7,10,12, 13, 16, 18, 53]. Interestingly, there were instances where multiple tools would identify a taxon as significantly differentially abundant; however, the direction of the change was different. Conflicting findings of this nature are also frequently reported in microbiome literature. Hence, the choice of DA analysis tool may be the source of such discrepancies as well.

Whilst we overall advocate for the use of a consensus method, if a single method was to be used, we would encourage the use of a more conservative method such as ALDEx2, which, when compared to other tools independent of using counts as input for all tools, had a high percentage of DA taxa identified that were consensus, at the cost of a reduced number of differences. This aligns with the recommendations of previous research, which compared DA methods across multiple datasets, although ANCOM-BC2, LinDA and ZicoSeq were not included in that analysis [25]. However, particularly in smaller studies, the use of this tool alone will likely result in few identified differences. Therefore, we believe a consensus method that combines conservative tools, such as ALDEx2, with less conservative tools as in the present study, strikes a better balance between lowering the incidence of false discovery, whilst maintaining reasonable power to detect differences.

A final consideration is the use of count data or proportion data as input. In the present study, we were initially hesitant to use count data as input for all tools as the count data used in this study was an ‘approximate count’ calculated from the total library size rather than a count measurement as you would see in 16S rRNA gene amplicon sequencing studies. However, tools like ZicoSeq and LinDA, whilst they do allow for proportion data as input, employ a different method for zero-handling when count data is used to allow for differences in library size to be accounted for and, so, are more robust [26,47]. Tools such as ANCOM-BC2, ALDEx2 and DESeq2 also require count inputs for this reason [42,45, 46]. Hence, we included results from both in the present study.

### Limitations

As a large portion of the sequenced reads were discarded during quality control due to being of human origin, sequencing depth was relatively low, and a strong linear correlation between sequencing depth and richness was observed, suggesting that the full diversity of the saliva and buccal microbiomes has not been captured. Differences associated with T2DM were exclusively in low-abundance taxa, and additional differences in other low-abundance species may have been missed due to the relatively low sequencing depth. Additionally, the difference in sequencing depth in swab samples may have influenced the findings. The sample size, particularly for T2DM comparisons, was also relatively small, so we may have insufficient power to detect differences. Therefore, there is a need for our results to be validated in future studies with a larger cohort and sequencing depth.

As MetaPhlAn4 determines taxonomy using multiple marker genes, the default output is relative abundance rather than counts, and it is not possible to get a count value as you would for 16S rRNA gene amplicon sequencing. In this study, for tools that required count input, approximate counts were estimated by multiplying relative abundance by total library size for each sample; however, this almost certainly overestimates the number of reads mapped to each taxon, as it does not account for reads that would not have mapped to any reference genomes. This may have unknown implications for results from differential abundance analysis tools that require count inputs to make inferences related to varying sequencing depth between samples. Using approximate counts rather than proportions as input for MaAsLin2, LinDA and ZicoSeq does somewhat alter the findings, though with most consensus taxa still identified as consensus.

Due to the low sequencing depth post-quality control, we did not attempt taxonomy analysis using metagenome-assembled genomes or strain-level analysis, which can improve the resolution of taxonomic and functional profiles. Future studies should consider the use of these over the use of a tool such as MetaPhlAn4.

Our study did not collect information associated with the participants’ oral health, which is known to influence the oral microbiome. Whilst participants were instructed to refrain from eating or drinking from 1 h prior to collection, they were not explicitly instructed to not brush their teeth or use mouthwash, which may influence the results. Additionally, the samples collected in the present study do not capture the full diversity of all oral microbial niches, and alternate sampling such as subgingival plaque may reveal differences not captured by the present study, particularly as subgingival plaque is frequently associated with periodontal health [62].

There was high metformin use in the present study, which may influence our findings. Metformin has previously been suggested to alter the gut microbiome composition [63]. Whilst this is generally thought to be due to the poor absorption of and accumulation of metformin in the gut, these effects may extend to the oral microbiome [64]. Unfortunately, due to the small number of participants with T2DM who did not use metformin, this could not be adjusted for statistically, and hence, future studies should take this into consideration.

Whilst this study focuses on the choice of DA analysis method and its impact on findings, there are many other factors that may influence microbiome study findings. These include, but are not limited to, sample storage method, choice of extraction method, sequencing type and taxonomic classification method, all of which should be considered when designing future studies [65].

## Conclusion

To our knowledge, this was the first study examining the oral microbiome in pregnant women with pre-existing T2DM. Overall, we identified comparably few differences in the oral microbiome between pregnant women with and without pre-existing T2DM, with a small number of taxonomy differences identified only at the late time point. Whilst the sample size and low sequencing depth may limit our ability to detect differences in the present study, the highly variable findings in previous research examining T2DM, GDM and the oral microbiome, and the few differences found in the present study, suggest that the true number of differentially abundant oral taxa due to diabetes mellitus is low. However, what this means for the impact of the oral microbiome on T2DM risk is not clear, and further research is needed to establish the existence of a causal relationship between the oral microbiome and health. Future studies should consider employing a consensus approach to DA analysis to improve confidence in reported findings.

## Supplementary material

10.1099/mgen.0.001385 Uncited Supplementary Material 1.

10.1099/mgen.0.001385 Uncited Supplementary Material 2.

## Abbreviations

AST: arcsine square root transformation
BMI: body mass index
BP: blood pressure
CLR: centred log-ratio
DA: differential abundance
GDM: gestational diabetes mellitus
HbA1c: haemoglobin A1c
HDL: high-density lipoprotein
IQR: interquartile range
LDL: low-density lipoprotein
OSCC: oral squamous cell carcinoma
PCOS: polycystic ovarian syndrome
RPK: reads per kilobase
SGBs: species genome bins
T2DM: type 2 diabetes mellitus
