# Supplementary material for: Consensus approach to differential abundance analysis detects few differences in the oral microbiome of pregnant women due to pre-existing type 2 diabetes mellitus
Source: Microb Genom. 2025 Apr 15;11(4):001385. doi: 10.1099/mgen.0.001385 (PMC12282217; doi:10.1099/mgen.0.001385)
Supplement: Uncited Supplementary Material 1. [file mgen-11-01385-s001.pdf]

Supplementary Figures

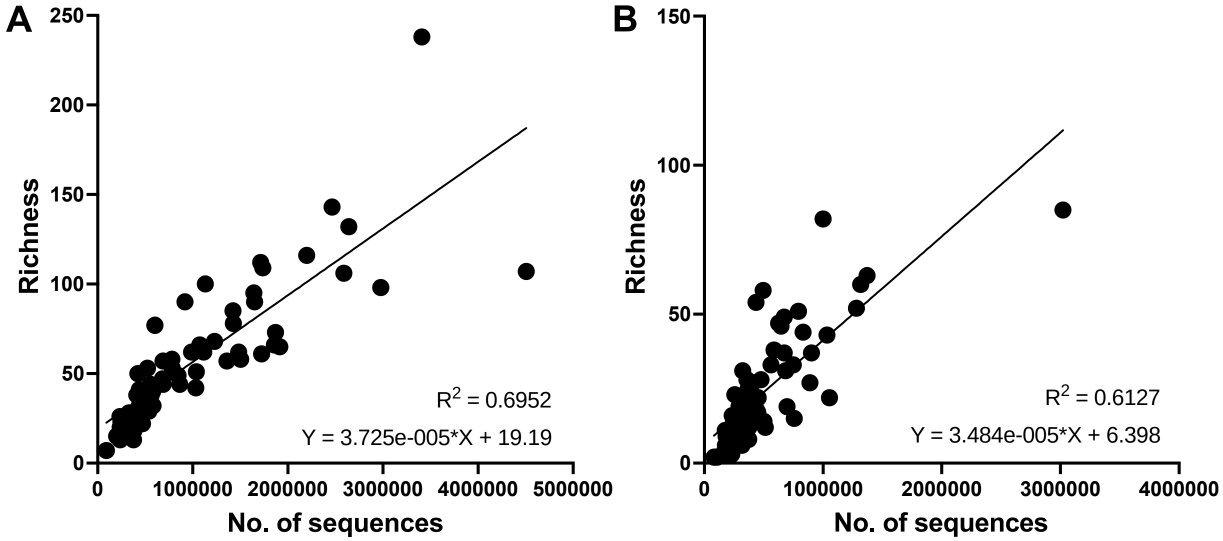

Figure S1: Relationship between number of sequences per sample and richness in **A)** rinse (n = 72) and **B)** swab (n = 72) samples.

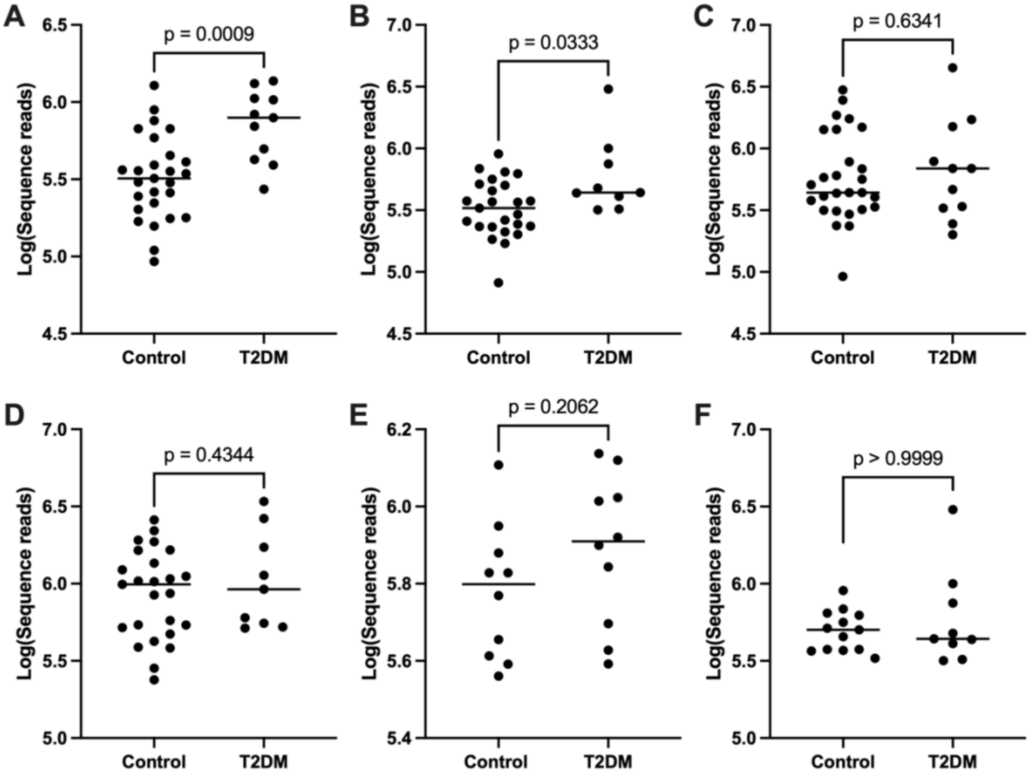

Figure S2: Sequencing depth of normoglycaemic controls vs T2DM swab samples at the early **(A)** and late **(B)** timepoints, rinse samples at the early **(C)** and late **(D)** timepoints, and subset of swab samples for investigation of effect of sequencing depth at the early **(E)** and late **(F)** timepoints. If data was normally distributed displayed p-value was determined by paired T-test, otherwise was determined by Wilcoxon test. Horizontal bar represents median.

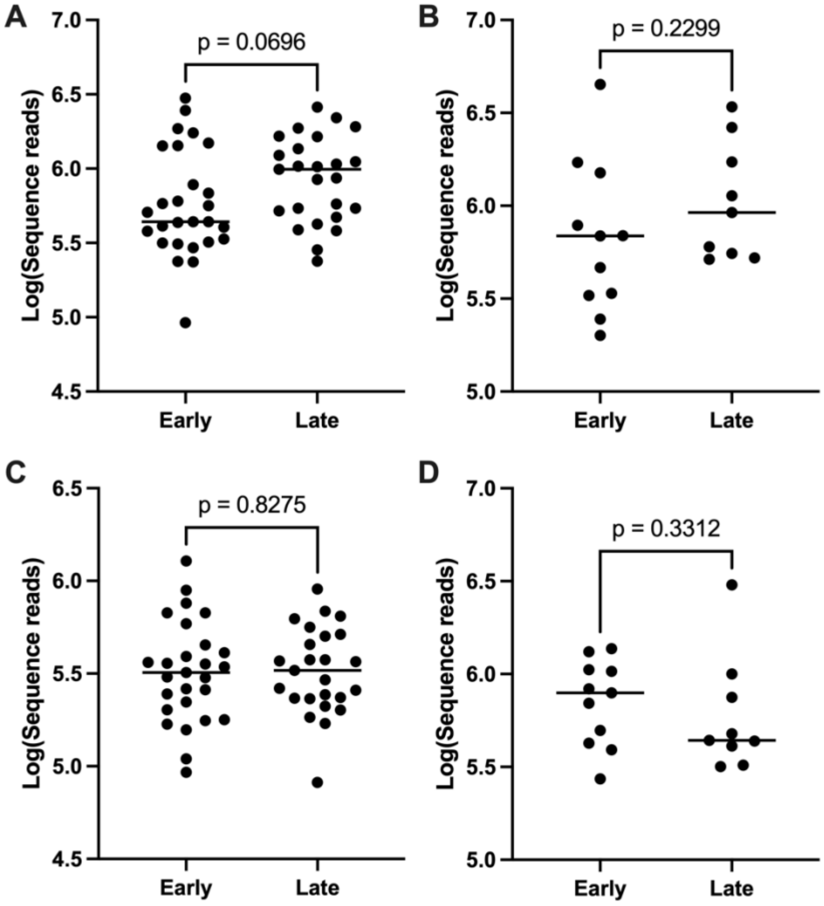

Figure S3: Comparing sequencing depth at the early and late timepoint in saliva rinse samples of **A)** normoglycaemic controls and **B)** T2DM, and in buccal swab samples of **C)** normoglycaemic controls and **D)** T2DM. If data was normally distributed displayed p-value was determined by paired T-test, otherwise was determined by Wilcoxon test. Horizontal bar represents median.

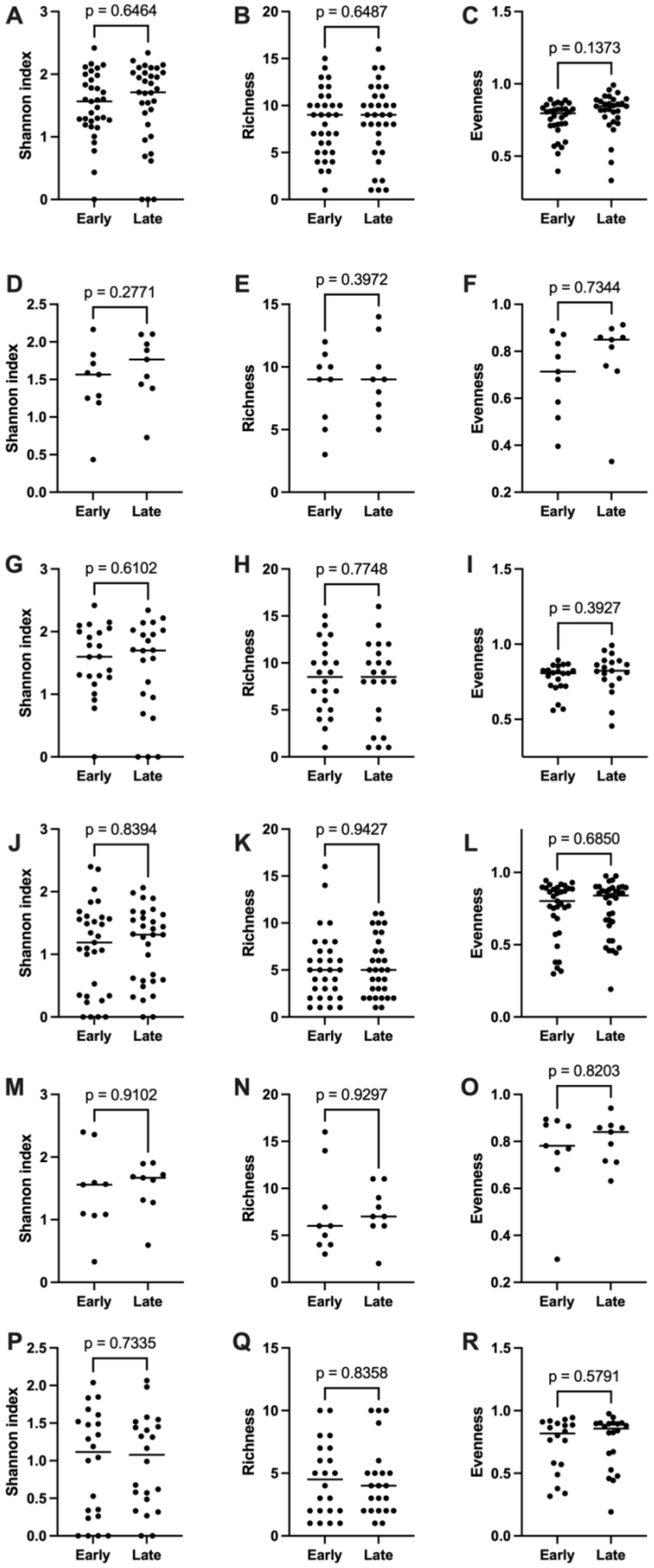

Figure S4: Alpha diversity of rarefied dataset across timepoints (Early: 26-28 weeks, Late: 34 - 38 weeks) as measured by Shannon Index (left), Richness (middle), and Evenness (right). **A-C:** Rinse samples across timepoints in whole cohort. **D-F:** Rinse samples across timepoints in T2DM participants only. **G-I:** Rinse samples across timepoints in normoglycaemic participants only. **J-L:** Swab samples across timepoints in whole cohort. **M-O:** Swab samples across timepoints in T2DM participants only. **P-R:** Swab samples across timepoints in normoglycaemic participants only. If data was normally distributed displayed p-value was determined by paired T-test, otherwise was determined by Wilcoxon test. Horizontal bar represents median.

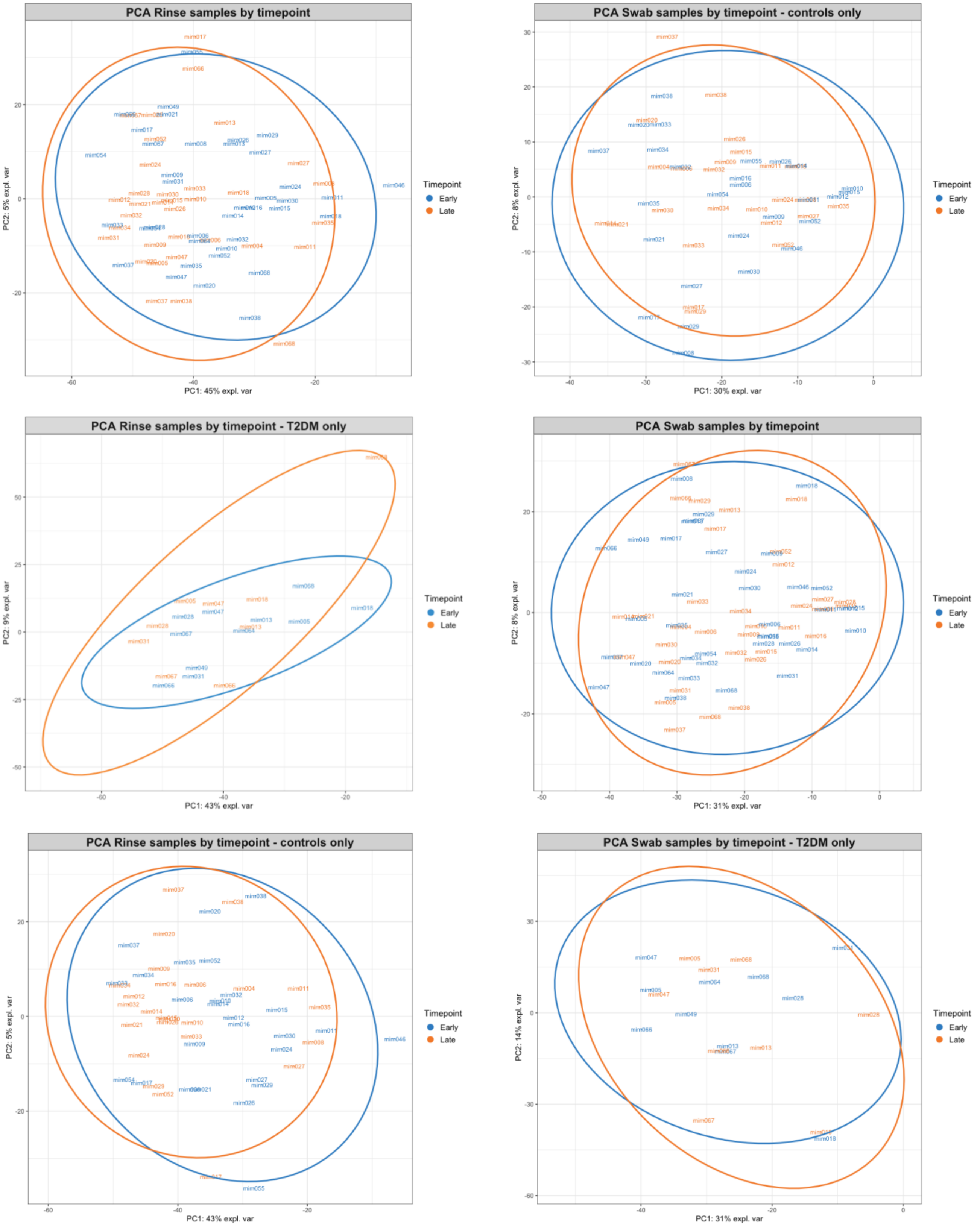

Figure S5: PCA of Aitchison distances from taxonomic profiles between early and late timepoints across pregnancy in saliva rinse **(A-C)** and buccal swab **(D-F)** samples**: A,D)** whole cohort, **B,D)** controls only, **C, F)** T2DM only

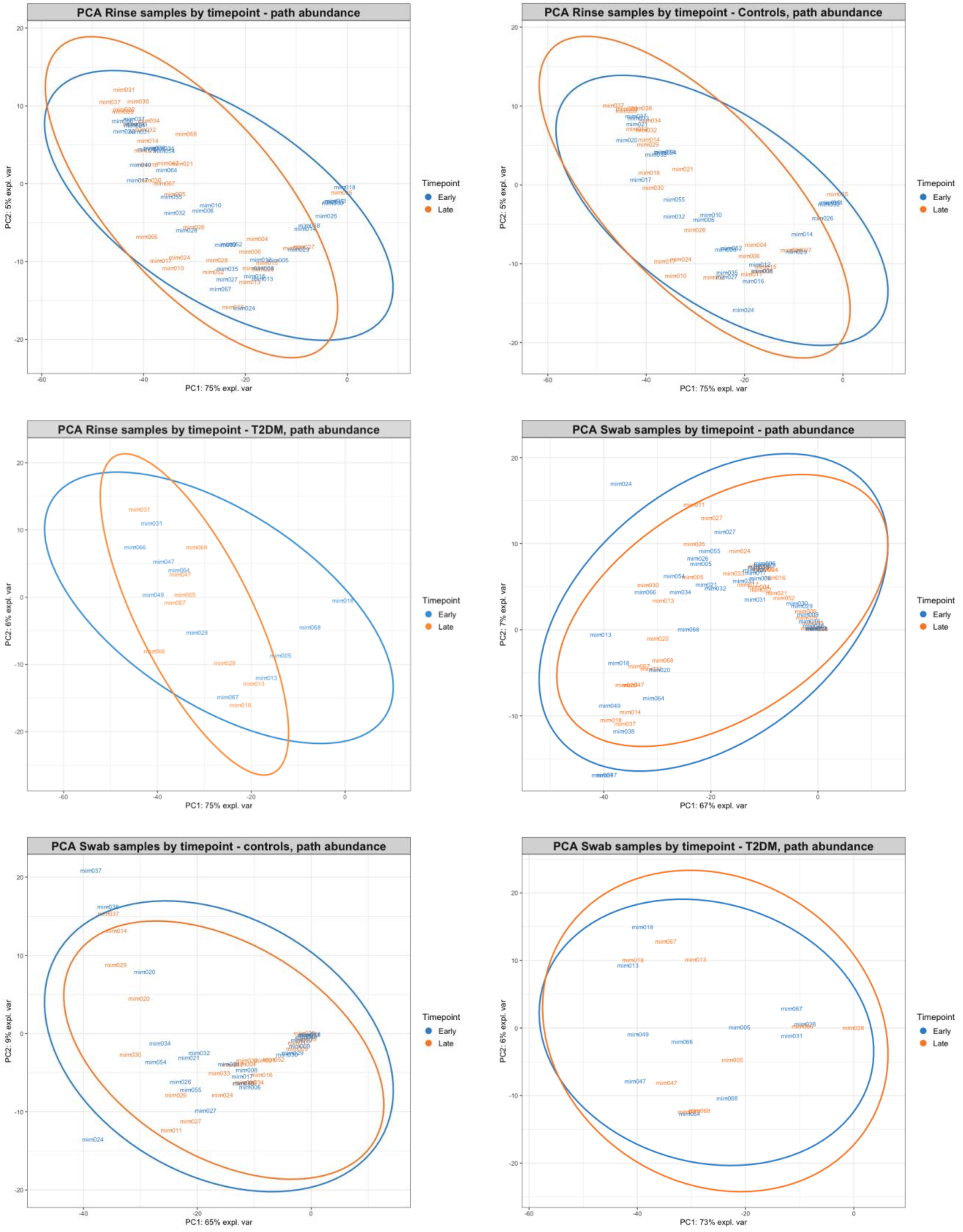

Figure S6: PCA of Aitchison distances from functional profiles between early and late timepoints across pregnancy in saliva rinse **(A-C)** and buccal swab **(D-F)** samples**: A,D)** whole cohort, **B,D)** controls only, **C, F)** T2DM only

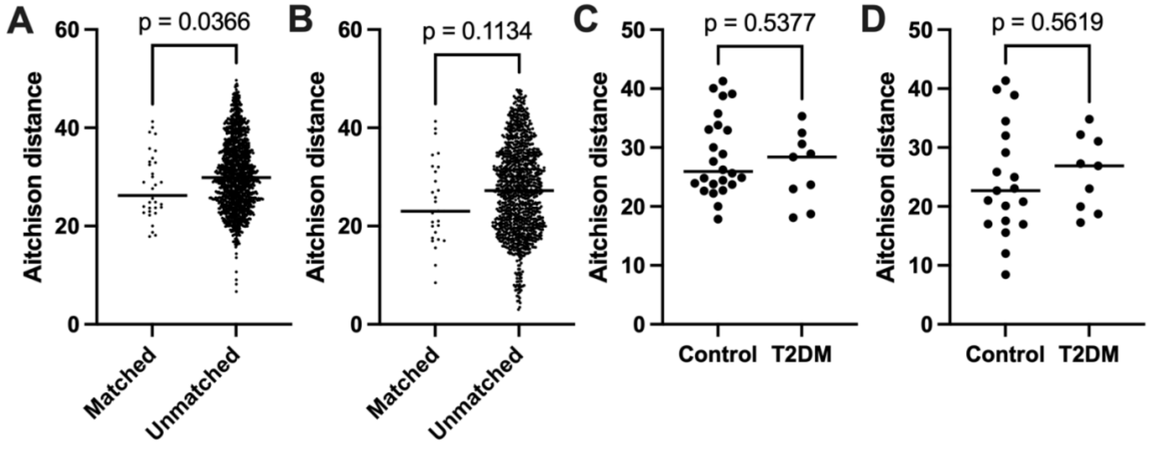

Figure S7: Aitchison distances from functional profiles between matched and unmatched oral microbiome CLR transformed data across timepoints in **A)** rinse and **B)** swab samples. Aitchison distances between matched samples across time points in normoglycemic controls and T2DM women in **C)** rinse and **D)** swab. If data was normally distributed displayed p-value was determined by paired T-test, otherwise was determined by Wilcoxon test. Horizontal bar represents median.

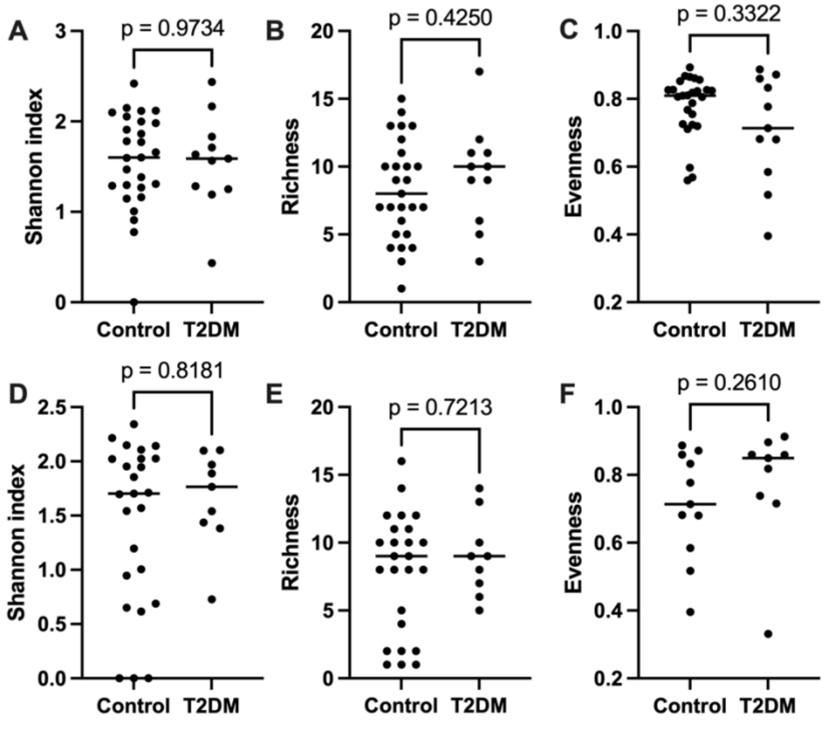

Figure S8: Alpha diversity of rarefied saliva rinse samples at early **(A-C)** and late **(D-F)** timepoints measured by Shannon Index (**A, D**), Richness (**B, E),** and Evenness (**C, F).** If data was normally distributed p-values were calculated by unpaired t-test, else they were calculated with the Mann-Whitney test.

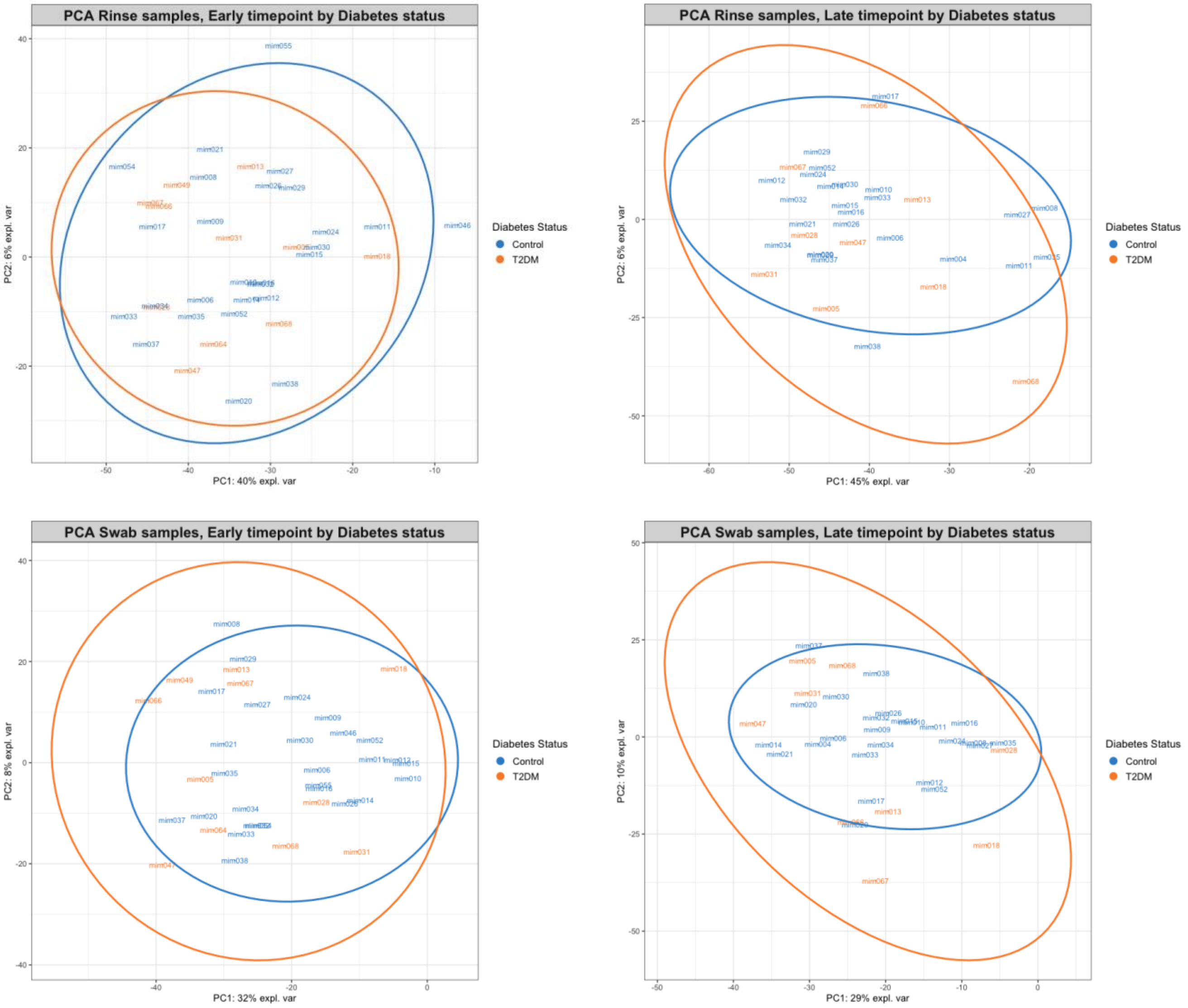

Figure S9: PCA of Aitchison distances from taxonomic profiles between T2DM and normoglycaemic controls in saliva rinse samples at early (**A**) and late (**B**) timepoints, and in buccal swab samples at early (**C**) and Late (**D**) timepoints.

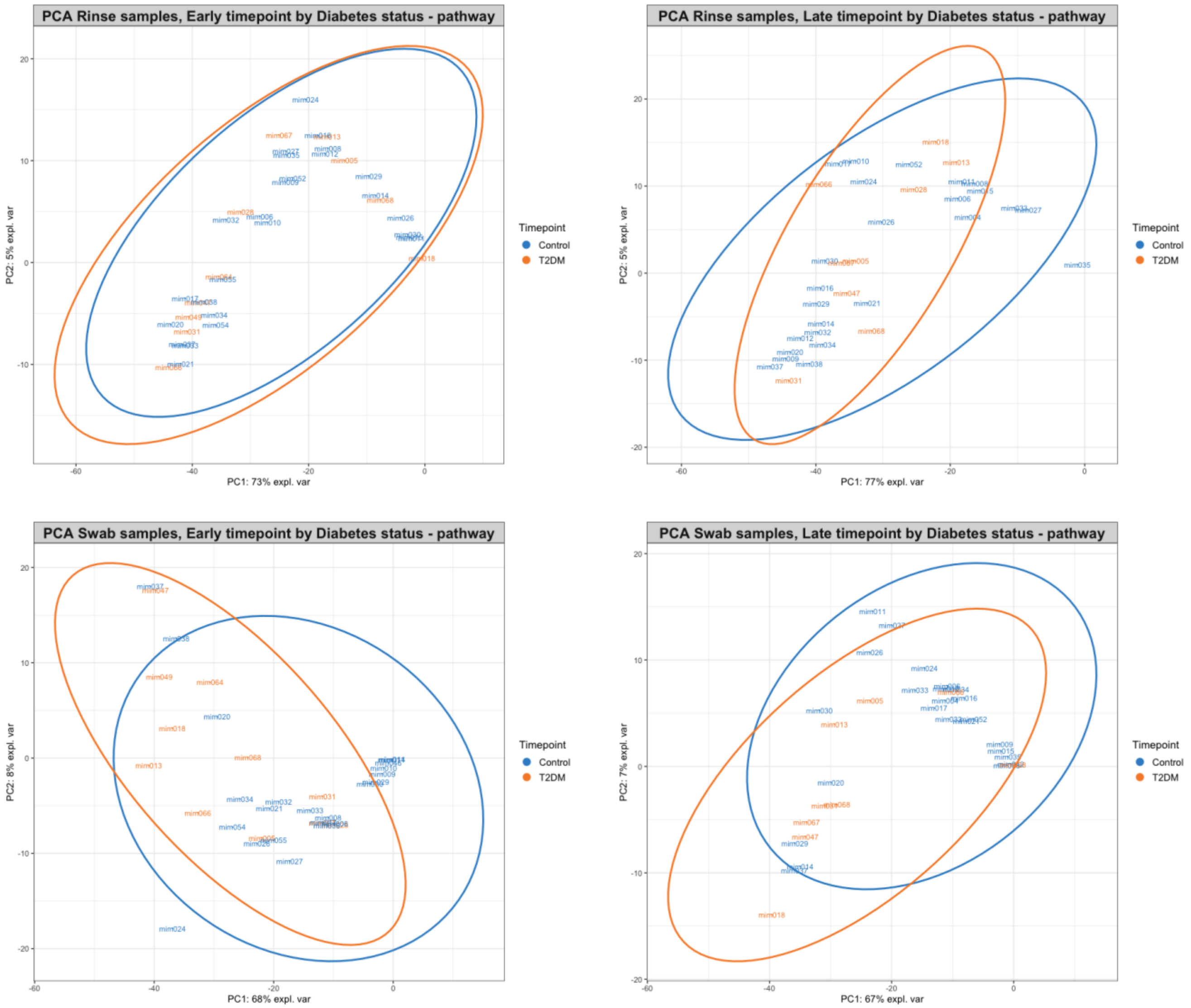

Figure S10: PCA of Aitchison distances from functional profiles between T2DM and normoglycaemic controls in saliva rinse samples at early (**A**) and late (**B**) timepoints, and in buccal swab samples at early (**C**) and Late (**D**) timepoints.

Supplementary Tables

Table S8: Significantly differentially abundant species between sample types detected by ≥3 DA analysis tools at the early timepoint.	
**Rinse samples**	**Swab Samples**
**Identified by all tools**	
Veillonella atypica SGB6936*	GGB10485 SGB49305 (*Enterococcaceae)**
**Identified by 5 tools**	
Prevotella pallens SGB1564[13456]*<br>GGB9707 SGB1522 (*Oscillospiraceae*)[12456]*	*Actinomyces oris* SGB15878[13456]*<br>*Streptococcus gordonii* GB8053[13456]*
**Identified by 4 tools**	
Aggregatibacter sp 2125159857 SGB9733[1456]*<br>*Campylobacter concisus* SGB19351[1456]<br>GGB1144 SGB1468 (*Prevotellaceae*)[1456]*<br>*Lancefieldella parvula* SGB966[1456]<br>*Megasphaera micronuciformis* SGB5868[1456]<br>*Prevotella jejuni* SGB1545[1456]*<br>*Prevotella melaninogenica* SGB1552[1456]*<br>*Prevotella nigrescens* SGB1561[1456]<br>*Prevotella oris* SGB1525[1456]*<br>*Prevotella vespertina* SGB1542[1456]*<br>*Veillonella dispar* SGB6952[1456]*<br>*Veillonella parvula* SGB6939[1456]*<br>*Veillonella rogosae* SGB6956[1456]*	
**Identified by 3 tools**	
Alloprevotella SGB1483[146]<br>*Alloprevotella* SGB1484[146]<br>*Eubacterium sulci* SGB3934[146]	

Fusobacterium nucleatum SGB6014[146]<br>*Gemella morbillorum* SGB7295[146]<br>GGB 1202 SGB1566[146] (*Bacteroidetes*)<br>*Granulicatella* SGB8255[145]*<br>*Neisseria subflava* SGB9450[145]*<br>*Prevotella conceptionensis* SGB1458[146]<br>*Prevotella denticola* SGB1540[145]<br>*Prevotella histicola* SGB1543[156]*<br>*Prevotella veroralis* SGB1541[146]<br>*Streptococcus mitis* SGB8168[156]<br>TM7 phylum sp oral taxon 352 SGB19860*<br>(*Candidatus Saccharibacteria* unclassified)[156]	
P-values are included in Table S6. Superscript represents tool: MaAsLin2[1], ANCOM-BC2[2], ALDEx2[3], DESeq2[4], LinDA[5] and ZicoSeq[6]. *, also identified when count inputs used for MaAsLin2, LinDA and ZicoSeq (Table S22)	

Table S9: Significantly differentially abundant genera between sample types detected by ≥3 DA analysis tools at the early timepoint.

**Rinse samples**	**Swab Samples**
**Identified by all tools**	
	GGB10485*
**Identified by 5 tools**	
Veillonella[12456]*	*Streptococcus*[13456]*
**Identified by 4 tools**	
Prevotella[1245]*<br>*Lancefieldella*[1456]*<br>GGB1144[1456]*<br>*Candidatus Saccharibacteria* unclassified[1456]*<br>*Megasphaera*[1456]*<br>*Neisseria*[1456]*<br>*Aggregatibacter*[1456]*	
**Identified by 3 tools**	

GGB1202[146]<br>*Granulicatella*[145]*<br>*Alloprevotella*[145]*	
P-values are included in Table S6. Superscript represents tool: MaAsLin2[1], ANCOM-BC2[2], ALDEx2[3], DESeq2[4], LinDA[5] and ZicoSeq[6]. *, also identified when count inputs used for all tools (Table S23).	

**Table S10:** Significantly differentially abundant family between sample types detected by ≥3 DA analysis tools at the early timepoint.	
**Rinse samples**	**Swab Samples**
**Identified by all tools**	
	*Enterococcaceae**
**Identified by 5 tools**	
Veillonellaceae[12456]*	*Streptococcaceae*[13456]*
**Identified by 4 tools**	
Prevotellaceae[1245]*<br>*Candidatus Saccharibacteria* unclassified[1456]*	
**Identified by 3 tools**	
Atopobiaceae[146]<br>FGB570[146]<br>*Carnobacteriaceae*[145]*	
P-values are included in Table S6. Superscript represents tool: MaAsLin2[1], ANCOM-BC2[2], ALDEx2[3], DESeq2[4], LinDA[5] and ZicoSeq[6]. *, also identified when count inputs used for all tools (Table S24).	

**Table S11:** Significantly differentially abundant order between sample types detected by ≥3 DA analysis tools at the early timepoint.	
**Rinse samples**	**Swab Samples**
**Identified by 5 tools**	
Bacteroidales[12456]*	*Lactobacillales*[13456]*
**Identified by 4 tools**	
Veillonellales[1245]*	
**Identified by 3 tools**	
OFGB570[146]<br>*Candidatus Saccharibacteria* unclassified[146]*<br>*Campylobacterales*[146]	
P-values are included in Table S6. Superscript represents tool: MaAsLin2[1], ANCOM-BC2[2], ALDEx2[3], DESeq2[4], LinDA[5] and ZicoSeq[6]. *, also identified when count inputs used for all tools (Table S25).	

**Table S12:** Significantly differentially abundant class between sample types detected by ≥3 DA analysis tools at the early timepoint.	
**Rinse samples**	**Swab Samples**
**Identified by all tools**	
	*Bacilli**
**Identified by 4 tools**	
Bacteroidia[1245]*<br>*Negativicutes*[1245]*<br>*Candidatus Saccharibacteria* unclassified[1456]	
**Identified by 3 tools**	
Coriobacteriia[146]<br>*Epsilonproetobacteria*[146]	
P-values are included in Table S6. Superscript represents tool: MaAsLin2[1], ANCOM-BC2[2], ALDEx2[3], DESeq2[4], LinDA[5] and ZicoSeq[6]. *, also identified when count inputs used for all tools (Table S26).	

**Table S13:** Significantly differentially abundant phylum between sample types detected by ≥3 DA analysis tools at the early timepoint.	
**Rinse samples**	**Swab Samples**
**Identified by 4 tools**	
Bacteroidetes[1245]*<br>*Candidatus Saccharibacteria*[1456]*	*Firmicutes*[1346]*
P-values are included in Table S6. Superscript represents tool: MaAsLin2[1], ANCOM-BC2[2], ALDEx2[3], DESeq2[4], LinDA[5] and ZicoSeq[6]. *, also identified when count inputs used for all tools (Table S27).	

**Table S14:** Significantly differentially abundant pathways between sample types by ≥ 3 DA analysis tools at the early timepoint.	
**Rinse samples**	**Swab Samples**
**Identified by 4 tools**	
1CMET2-PWY: Folate transformation III [1456]*	LACTOSECAT-PWY: Lactose degradation 1[1256]*
**Identified by 3 tools**	
PWY-7200: superpathway of pyrimidine deoxyribonucleoside salvage[156]*<br>PWY-5130: 2-oxobutanoate degradation I[156]<br>PWY-6609: adenine and adenosine salvage III[156]<br>PYRIDNUCSYN-PWY: NAD de novo biosynthesis[156]<br>PWY-7977: L-methionine biosynthesis IV[156]<br>PPGPPMET-PWY: ppGpp metabolism[156]	
P-values are included in Table S6. Superscript represents tool: MaAsLin2[1], ANCOM-BC2[2], ALDEx2[3], DESeq2[4], LinDA[5] and ZicoSeq[6]. *, also identified when count inputs used for MaAsLin2, LinDA and ZicoSeq (Table S28).	

Table S15: Significantly differentially abundant species between sample types detected by ≥3 DA analysis tools at the late timepoint.

Rinse samples	Swab Samples
**Identified by 5 tools**	
Veillonella atypica SGB6936[13456*]<br>*Veillonella dispar* SGB6952[13456*]<br>GGB1144 SGB1468[13456*]<br>*Prevotella jejuni* SGB1545[13456*]<br>*Prevotella pallens* SGB1564[13456*]<br>*Campylobacter concisus* SGB19351 group[13456*]<br>*Lancefieldella parvula* SGB966[12456*]<br>*Prevotella melaninogenica* SGB1552[12456*]<br>*Neisseria subflava* SGB9450[12456*]	GGB10485 SGB49305[13456*]
**Identified by 4 tools**	
Streptococcus parasanguinis SGB8071[3456*]<br>*Veillonella parvula* SGB6939[1345*]<br>TM7 phylum *sp.* Oral taxon 352 SGB19860[1456*]<br>*Granulicatella adiacens* SGB8249[1456]<br>*Granulicatella* SGB8255 group[1456*]<br>*Streptococcus infantis* SGB8095[1456*]<br>*Veillonella rogosae* SGB6956[1456]<br>*Megasphaera micronuciformis* SGB5868[1456*]<br>*Porphyromonas pasteri* SGB2043[1456*]<br>*Alloprevotella* SGB1484[1456*]<br>*Prevotella salivae* SGB1522[456*]<br>*Prevotella nanceiensis* SGB1536 group[1456]<br>*Prevotella oris* SGB1525[1456*]<br>*Prevotella histicola* SGB1543[1456*]<br>*Prevotella nigrescens* SGB1561[1456*]<br>*Fusobacterium nucleatum* SGB6007[1456*]<br>*Aggregatibacter* sp. 2125159857 SGB9733[1456]	*Streptococcus gordonii* SGB8053[3456*]<br>*Actinomyces gerencseriae* SGB15878[1456*]
**Identified by 3 tools**	
Actinomyces sp. ICM58 SGB17169[146]<br>*Actinomyces sp.* ICM47 SGB17167 group[456*]<br>*Solobacterium* SGB6829[145]<br>*Selenomonas* SGB5880[146]<br>*Eubacterium sulci* SGB3934[146]<br>*Tannerella forsythia* SGB2053[146]	*Streptococcus vestibularis* SGB8003[345*]

Alloprevotella SGB1483[146]<br>*Alloprevotella tannerae* SGB1411[146]<br>*Prevotella shahii* SGB1456[146]<br>*Prevotella conceptionensis* SGB1458[146]<br>*Kingella denitrificans* SGB9437[146]<br>*Kingella bonacorsii* SGB9422[146]<br>*Prevotella denticola* SGB1540[156]<br>*Prevotella vespertina* SGB1542[156]*<br>*Fusobacterium nucleatum* SGB6014[156]	
P-values are included in Table S7. Superscript represents tool: MaAsLin2[1], ANCOM-BC2[2], ALDEx2[3], DESeq2[4], LinDA[5] and ZicoSeq[6]. *, also identified when count inputs used for all tools (Table S29).	

**Table S16:** Significantly differentially abundant genera between sample types detected by ≥3 DA analysis tools at the late timepoint.	
**Rinse samples**	**Swab Samples**
**Identified by all tools**	
*Prevotella**	GGB10485*
**Identified by 5 tools**	
GGB1144[13456]*	*Streptococcus*[13456]*
**Identified by 4 tools**	
Lancefieldella[1456]*<br>*Alloprevotella*[1456]*<br>*Granulicatella*[1456]*<br>*Veillonella*[1456]*<br>*Fusobacterium*[1456]<br>*Kingella*[1456]*<br>*Neisseria*[1456]<br>*Campylobacter*[1456]*<br>*Aggregatibacter*[1456]	
**Identified by 3 tools**	
GGB12785[146]<br>*Candidatus Saccharibacteria* unclassified[146]*<br>*Dialister*[146]<br>*Parvimonas*[146]	

Porphyromonas[146]<br>*Megasphaera*[146]*	
P-values are included in Table S7. Superscript represents tool: MaAsLin2[1], ANCOM-BC2[2], ALDEx2[3], DESeq2[4], LinDA[5] and ZicoSeq[6]. *, also identified when count inputs used for all tools (Table S30).	

**Table S17:** Significantly differentially abundant family between sample types detected by ≥3 DA analysis tools at the late timepoint.	
**Rinse samples**	**Swab Samples**
**Identified by all tools**	
*Prevotellaceae**	*Enterococcaceae**
**Identified by 5 tools**	
	Streptococcaceae[13456]*
**Identified by 4 tools**	
Atopobiaceae[1456]*<br>*Porphyromonadaceae*[1456]*<br>*Carnobacteriaceae*[1456]*<br>*Veillonellaceae*[1456]*<br>*Neisseriaceae*[1456]*<br>*Campylobacteraceae*[1456]*	
**Identified by 3 tools**	
FGB570[146]<br>*Weeksellaceae*[146]<br>*Candidatus Nanoglingivalaceae*[146]<br>*Candidatus Saccharibacteria* unclassified[146]<br>*Peptoniphilaceae*[146]<br>*Fusobacteriaceae*[146]*	
P-values are included in Table S7. Superscript represents tool: MaAsLin2[1], ANCOM-BC2[2], ALDEx2[3], DESeq2[4], LinDA[5] and ZicoSeq[6]. *, also identified when count inputs used for all tools (Table S31).	

Table S18: Significantly differentially abundant order between sample types detected by ≥3 DA analysis tools at the late timepoint.

**Rinse samples**	**Swab Samples**
**Identified by 5 tools**	
Bacteroidales[12456]*	*Lactobacillales*[13456]*
**Identified by 4 tools**	
Veillonellales[1256]*<br>*Coriobacteriales*[1456]*<br>*Neisseriales*[1456]*<br>*Campylobacterales*[1456]*	
**Identified by 3 tools**	
OFGB570[146]<br>*Candidatus Nanogingivales*[146]<br>*Candidatus Saccharibacteria* unclassified[146]<br>*Eubacteriales*[146]*<br>*Tissierellales*[146]	

P-values are included in Table S7. Superscript represents tool: MaAsLin2[1], ANCOM-BC2[2], ALDEx2[3], DESeq2[4], LinDA[5] and ZicoSeq[6]. *, also identified when count inputs used for all tools (Table S32).

Table S19: Significantly differentially abundant class between sample types detected by ≥3 DA analysis tools at the late timepoint.

Rinse samples	Swab Samples
**Identified by 5 tools**	
Bacteroidia[12456]*	*Bacilli*[13456]*
**Identified by 4 tools**	
Negativicutes[1246]*<br>*Tissierellia*[1246]*<br>*Coriobacteriia*[1456]*<br>*Epsilonproteobacteria*[1456]*	
**Identified by 3 tools**	
CFGB570[146]<br>*Candidatus Nanosyncoccalia*[146]<br>*Candidatus Saccharibacteria* unclassified[146]*<br>*Clostridia*[146]*<br>*Betaproteobacteria*[146]	

P-values are included in Table S7. Superscript represents tool: MaAsLin2[1], ANCOM-BC2[2], ALDEx2[3], DESeq2[4], LinDA[5] and ZicoSeq[6]. *, also identified when count inputs used for all tools (Table S33).

Table S20: Significantly differentially abundant phylum between sample types detected by ≥3 DA analysis tools at the late timepoint.

Rinse samples	Swab Samples
**Identified by 5 tools**	
Bacteroidetes[12456]*	
**Identified by 4 tools**	
Candidatus Saccharibacteria[1456]*	*Firmicutes*[1345]*
**Identified by 3 tools**	
Proteobacteria[156]	

P-values are included in Table S7. Superscript represents tool: MaAsLin2[1], ANCOM-BC2[2], ALDEx2[3], DESeq2[4], LinDA[5] and ZicoSeq[6]. *, also identified when count inputs used for all tools (Table S34).

Table S21: Significantly differentially abundant pathways between sample types detected by ≥3 DA analysis tools at the late timepoint.

**Rinse samples**	**Swab Samples**
**Identified by all tools**	
GLUCONEO-PWY*<br>PYRIDNUCSYN-PWY*	
**Identified by 5 tools**	
1CMET2-PWY[13456]*<br>HEMESYN2-PWY[13456]*<br>PWY-7200[13456]*<br>PWY-7953[13456]*<br>RIBOSYN2-PWY[12456]*	
**Identified by 4 tools**	
PWY-5973[1246]*<br>GLYCOLYSIS[1246]*<br>PEPTIDOGLYCANSYN-PWY[1246]*<br>PWY-5484[1246]*<br>PWY-5837[1256]<br>PWY-6387[1246]*<br>PWY-6700[1246]*<br>ANAEROFRUCAT-PWY[1456]<br>CALVIN-PWY[1456]<br>COLANSYN-PWY[1456]*<br>HISDEG-PWY[1456]<br>HISTSYN-PWY[1456]<br>NONMEVIPP-PWY[1456]<br>PANTOSYN-PWY[1456]<br>PPGPPMET-PWY[1456]*<br>PWY-1269[1456]<br>PWY-241[1456]*<br>PWY-5030[1456]<br>PWY-5913[1456]<br>PWY-7117[1456]<br>PWY-7977[1456]<br>PWY0-1241[1456]<br>PWY0-1261[1456]<br>SER-GLYSYN-PWY[1456]<br>THISYNARA-PWY[1456]	VALSYN-PWY[1356]*

**Identified by 3 tools**	
PWY-5897[126]<br>PWY-5898[126]<br>PWY-5899[126]<br>PWY66-429[124]*<br>ARGYSYN-PWY[146]<br>ARGYSYNBSUB-PWY[146]<br>COA-PWY-1[146]<br>COA-PWY[146]<br>COMPLETE-ARO-PWY[146]<br>DAPLYSINESYN-PWY[146]<br>FASYN-ELONG-PWY[146]<br>GLUTORN-PWY[146]<br>HEME-BIOSYNTHESIS-II[146]<br>HOMOSER-METSYN-PWY[146]<br>MET-SAM-PWY[146]<br>METSYN-PWY[146]<br>NAGLIPASYN-PWY[146]<br>OANTIGEN-PWY[146]<br>PHOSLIPSYN-PWY[146]<br>PWY-2942[146]<br>PWY-3841[146]<br>PWY-4041[146]<br>PWY-5097[146]<br>PWY-5188[146]<br>PWY-5347[146]<br>PWY-5918[146]<br>PWY-5989[146]<br>PWY-6147[146]<br>PWY-6282[146]<br>PWY-6385[146]<br>PWY-6386[146]<br>PWY-6628[146]<br>PWY-6630[146]<br>PWY-6897[146]<br>PWY-724[146]<br>PWY-7282[146]<br>PWY-7328[146]	PWY-5676[235]*

PWY-7345[146]<br>PWY-7560[146]<br>PWY-7664[146]<br>PWY-7851[146]<br>PWY-7858[146]<br>PWY-8004[146]<br>PWY-8073[146]<br>PWY0-1061[146]<br>TRNA-CHARGING-PWY[146]<br>HEME-BIOSYNTHESIS-II-1[156]<br>P42-PWY[156]<br>PWY-5005[156]<br>PWY-5130[156]<br>PWY-5855[156]<br>PWY-6608[156]<br>PWY-6803[156]<br>PWY0-1479[156]<br>PWY66-389[156]<br>PYRIDOXSYN-PWY[156]	
P-values are included in Table S7. Superscript represents tool: MaAsLin2[1], ANCOM-BC2[2], ALDEx2[3], DESeq2[4], LinDA[5] and ZicoSeq[6]. *, also identified when count inputs used for all tools (Table S35).	

**Table S22:** Significantly differentially abundant species between sample types detected by ≥3 DA analysis tools at the early timepoint when counts are used as input for all tools.	
**Rinse samples**	**Swab Samples**
**Identified by all tools**	
	GGB10485 SGB49305*
**Identified by 5 tools**	
Veillonella atypica SGB6936[13456]*<br>*Prevotella salivae* SGB1522[12456]*	*Actinomyces oris* SGB15878[13456]*<br>*Streptococcus gordonii* SGB8053[13456]*
**Identified by 4 tools**	
Aggregatibacter sp. 2125159857 SGB9733[1456]*<br>GGB1144 SGB1468[1456]*<br>*Prevotella vespertina* SGB1542[1456]*<br>*Prevotella pallens* SGB1564[1345]*	*Actinomyces oris* SGB15878[1345]*
**Identified by 3 tools**	
Prevotella melaninogenica SGB1552[145]*<br>*Veillonella rogosae* SGB6956[145]*<br>*Prevotella jejuni* SGB1545[145]*<br>*Prevotella histicola* SGB1543[156]*<br>*Veillonella parvula* SGB6939[145]*<br>*Veillonella dispar* SGB6952[145]*<br>*Neisseria subflava* SGB9450[145]*<br>*Granulicatella* SGB8255[145]*<br>TM7 phylum sp. Oral taxon 352 SGB19860[156]*<br>*Prevotella oris* SGB1525[145]*	*Leptotrichia hongkongensis* SGB6059[125]<br>*Lautropia dentalis* SGB13164[135]
P-values are included in Table S6. Superscript represents tool: MaAsLin2[1], ANCOM-BC2[2], ALDEx2[3], DESeq2[4], LinDA[5] and ZicoSeq[6]. *, also identified when proportion inputs used for MaAsLin2, LinDA and ZicoSeq (Table S8).	

Table S23: Significantly differentially abundant genera between sample types detected by ≥3 DA analysis tools at the early timepoint when counts are used as input for all tools.

**Rinse samples**	**Swab Samples**
**Identified by all tools**	
	GGB10485*
**Identified by 4 tools**	
Prevotella[1245]*<br>*Veillonella*[1245]*<br>*Aggregatibacter*[1456]*<br>*Fusobacterium*[1456]<br>*Candidatus Saccharibacteria* unclassified[1456]*	
**Identified by 3 tools**	
Lancefieldella[456]*<br>GGB1144[145]*<br>*Granulicatella*[145]*<br>*Neisseria*[145]*<br>*Alloprevotella*[456]*<br>*Megasphaera*[456]*	*Streptococcus*[246]*

P-values are included in Table S6. Superscript represents tool: MaAsLin2[1], ANCOM-BC2[2], ALDEx2[3], DESeq2[4], LinDA[5] and ZicoSeq[6]. *, also identified when proportion inputs used for MaAsLin2, LinDA and ZicoSeq (Table S9).

Table S24: Significantly differentially abundant family between sample types detected by ≥3 DA analysis tools at the early timepoint when counts are used as input for all tools.

Rinse samples	Swab Samples
**Identified by all tools**	
	*Enterococcaceae**
**Identified by 5 tools**	
Veillonellaceae[12456]*	
**Identified by 4 tools**	
Prevotellaceae[1245]*<br>*Candidatus Saccharibacteria* unclassified[1456]*	*Streptococcaceae*[1346]*
**Identified by 3 tools**	
Fusobacteriaceae[145]<br>*Carnobacteriaceae*[145]*<br>*Neisseriaceae*[145]<br>*Pasteurellaceae*[145]	

P-values are included in Table S6. Superscript represents tool: MaAsLin2[1], ANCOM-BC2[2], ALDEx2[3], DESeq2[4], LinDA[5] and ZicoSeq[6]. *, also identified when proportion inputs used for MaAsLin2, LinDA and ZicoSeq (Table S10).

Table S25: Significantly differentially abundant order between sample types detected by ≥3 DA analysis tools at the early timepoint when counts are used as input for all tools.

Rinse samples	Swab Samples
**Identified by 5 tools**	
Veillonellales[12456]*<br>*Bacteroidales*[12456]*	
**Identified by 4 tools**	
	Lactobacillales[1345]*
**Identified by 3 tools**	
Candidatus Saccharibacteria unclassified[456]*<br>*Fusobacteriales*[456]	

P-values are included in Table S6. Superscript represents tool: MaAsLin2[1], ANCOM-BC2[2], ALDEx2[3], DESeq2[4], LinDA[5] and ZicoSeq[6]. *, also identified when proportion inputs used for MaAsLin2, LinDA and ZicoSeq (Table S10).

Table S26: Significantly differentially abundant class between sample types detected by ≥3 DA analysis tools at the early timepoint when counts are used as input for all tools.

Rinse samples	Swab Samples
**Identified by 5 tools**	
	Bacilli[12345]*
**Identified by 3 tools**	
Bacteroidia[124]*<br>*Negativicutes*[246]*	*Candidatus Saccharimonia*[125]

P-values are included in Table S6. Superscript represents tool: MaAsLin2[1], ANCOM-BC2[2], ALDEx2[3], DESeq2[4], LinDA[5] and ZicoSeq[6]. *, also identified when proportion inputs used for MaAsLin2, LinDA and ZicoSeq (Table S11).

Table S27: Significantly differentially abundant phylum between sample types detected by ≥3 DA analysis tools at the early timepoint when counts are used as input for all tools.

Rinse samples	Swab Samples
**Identified by 5 tools**	
Bacteroidetes[12456]*	
**Identified by 4 tools**	
Fusobacteria[1456]<br>*Candidatus Saccharibacteria*[1456]*	
**Identified by 3 tools**	
	Firmicutes[134]*

P-values are included in Table S6. Superscript represents tool: MaAsLin2[1], ANCOM-BC2[2], ALDEx2[3], DESeq2[4], LinDA[5] and ZicoSeq[6]. *, also identified when proportion inputs used for MaAsLin2, LinDA and ZicoSeq (Table S13).

**Table S28:** Significantly differentially abundant pathways between sample types detected by ≥3 DA analysis tools at the early timepoint when counts are used as input for all tools.	
**Rinse samples**	**Swab Samples**
**Identified by 4 tools**	
1CMET2-PWY[1456]*<br>PWY-7200[1456]*	LACTOSECAT-PWY[1256]*
**Identified by 3 tools**	
	PWY-5981[156]<br>PWY-7356[156]
P-values are included in Table S6. Superscript represents tool: MaAsLin2[1], ANCOM-BC2[2], ALDEx2[3], DESeq2[4], LinDA[5] and ZicoSeq[6]. *, also identified when proportion inputs used for MaAsLin2, LinDA and ZicoSeq (Table S14).	

**Table S29:** Significantly differentially abundant species between sample types detected by ≥3 DA analysis tools at the late timepoint when counts are used as input for all tools.	
**Rinse samples**	**Swab Samples**
**Identified by 5 tools**	
Veillonella atypica SGB6936[13456]*<br>GGB1144 SGB1468[13456]*<br>*Prevotella jejuni* SGB1545[13456]*<br>*Prevotella pallens* SGB1564[13456]*<br>*Lancefieldella parvula* SGB966[12456]*	GGB10485 SGB49305[13456]*
**Identified by 4 tools**	
Streptococcus parasanguinis SGB8071[1345]*<br>*Veillonella parvula* SGB6939[1345]*<br>*Veillonella dispar* SGB6952[1345]*<br>*Campylobacter concisus* SGB19351 group[1345]*<br>*Prevotella melaninogenica* SGB1552[1245]*<br>*Neisseria subflava* SGB9450[1245]*<br>*Prevotella histicola* SGB1543[1456]*<br>*Prevotella salivae* SGB1522[1456]*<br>*Megasphaera micronuciformis* SGB5868[1456]*	*Streptococcus gordonii* SGB8053[1345]*<br>*Streptococcus vestibularis* SGB8003[1345]*
**Identified by 3 tools**	
Prevotella nigrescens SGB1561[145]*<br>*Actinomyces sp. ICM47* SGB17167 group[145]*<br>*Granulicatella* SGB8255[145]*<br>*Streptococcus infantis* SGB8095[145]*<br>*Prevotella oris* SGB1525[145]*<br>*Prevotella vespertina* SGB1542[156]*<br>TM7 phylum *sp.* Oral taxon 352 SGB19860 group[145]*<br>*Porphyromonas pasteri* SGB2043[145]<br>*Alloprevotella* SGB1484[456]*<br>*Fusobacterium nucleatum* SGB6007[456]*	*Streptococcus peroris* SGB8084[135]<br>*Streptococcus mutans* SGB8000[135]<br>GGB79977 SGB1466[135]<br>*Capnocytophaga* SGB2480[135]<br>*Lautropia dentalis* SGB13164[135]<br>*Ottowia sp. Maseille P4747* SGB12664[135]<br>*Actinomyces oris* SGB15878[146]*
P-values are included in Table S7. Superscript represents tool: MaAsLin2[1], ANCOM-BC2[2], ALDEx2[3], DESeq2[4], LinDA[5] and ZicoSeq[6]. *, also identified when proportion inputs used for MaAsLin2, LinDA and ZicoSeq (Table S15).	

Table S30: Significantly differentially abundant genera between sample types detected by ≥3 DA analysis tools at the late timepoint when counts are used as input for all tools.

Rinse samples	Swab Samples
**Identified by all tools**	
	GGB10485*
**Identified by 5 tools**	
Prevotella[12345]*	*Streptococcus*[13456]*
**Identified by 4 tools**	
GGB1144[1345]*<br>*Campylobacter*[1456]*<br>*Lancefieldella*[1456]*	
**Identified by 3 tools**	
Alloprevotella[145]*<br>*Granulicatella*[145]*<br>*Megasphaera*[156]*<br>*Veillonella*[145]*<br>*Candidatus Saccharibacteria* unclassified[456]*<br>*Kingella*[456]*	*Arachnia*[135]<br>GGB79977[135]<br>*Ottowia*[135]

P-values are included in Table S7. Superscript represents tool: MaAsLin2[1], ANCOM-BC2[2], ALDEx2[3], DESeq2[4], LinDA[5] and ZicoSeq[6]. *, also identified when proportion inputs used for MaAsLin2, LinDA and ZicoSeq (Table S16).

**Table S31:** Significantly differentially abundant family between sample types detected by ≥3 DA analysis tools at the late timepoint when counts are used as input for all tools.	
**Rinse samples**	**Swab Samples**
**Identified by all tools**	
	*Enterococcaceae**
**Identified by 5 tools**	
Prevotellaceae[12345]*	*Streptococcaceae*[13456]*
**Identified by 4 tools**	
Atopobiaceae[1456]*	
**Identified by 3 tools**	
Carnobacteriaceae[145]*<br>*Campylobacteraceae*[145]*<br>*Porphyromonadaceae*[145]*<br>*Veillonellaceae*[145]*<br>*Neisseriaceae*[145]*<br>*Fusobacteriaceae*[145]*	*Propionibacteriaceae*[135]<br>FGB76125[135]<br>*Comamonadaceae*[135]
P-values are included in Table S7. Superscript represents tool: MaAsLin2[1], ANCOM-BC2[2], ALDEx2[3], DESeq2[4], LinDA[5] and ZicoSeq[6]. *, also identified when proportion inputs used for MaAsLin2, LinDA and ZicoSeq (Table S17).	

Table S32: Significantly differentially abundant order between sample types detected by ≥3 DA analysis tools at the late timepoint when counts are used as input for all tools.

Rinse samples	Swab Samples
**Identified by 5 tools**	
	Lactobacillales[13456]*<br>*Bacilli*[13456]
**Identified by 4 tools**	
Bacteroidales[1256]*<br>*Coriobacteriales*[1456]*<br>*Eubacteriales*[1456]*	
**Identified by 3 tools**	
Veillonellales[125]*<br>*Campylobacterales*[145]*<br>*Neisseriales*[145]*	*Propionibacteriales*[135]<br>OFGB76125[135]<br>CFGB76125[135]

P-values are included in Table S7. Superscript represents tool: MaAsLin2[1], ANCOM-BC2[2], ALDEx2[3], DESeq2[4], LinDA[5] and ZicoSeq[6]. *, also identified when proportion inputs used for MaAsLin2, LinDA and ZicoSeq (Table S18).

Table S33: Significantly differentially abundant class between sample types detected by ≥3 DA analysis tools at the late timepoint when counts are used as input for all tools.

Rinse samples	Swab Samples
**Identified by 5 tools**	
Bacteroidia[12456]*	*Bacilli*[13456]*
**Identified by 4 tools**	
Epsilonproteobacteria[1456]*<br>*Coriobacteriia*[1456]*<br>*Clostridia*[1456]*	
**Identified by 3 tools**	
Negativicutes[256]*<br>*Tissierellia*[246]*<br>*Candidatus Saccharibacteria* unclassified[456]*	CFGB76125[135]

P-values are included in Table S7. Superscript represents tool: MaAsLin2[1], ANCOM-BC2[2], ALDEx2[3], DESeq2[4], LinDA[5] and ZicoSeq[6]. *, also identified when proportion inputs used for MaAsLin2, LinDA and ZicoSeq (Table S19).

**Table S34:** Significantly differentially abundant phylum between sample types detected by ≥3 DA analysis tools at the late timepoint when counts are used as input for all tools.	
**Rinse samples**	**Swab Samples**
**Identified by 5 tools**	
Bacteroidetes[12456]*	
**Identified by 4 tools**	
Candidatus Saccharibacteria[1456]*	
**Identified by 3 tools**	
	Firmicutes[134]*
P-values are included in Table S7. Superscript represents tool: MaAsLin2[1], ANCOM-BC2[2], ALDEx2[3], DESeq2[4], LinDA[5] and ZicoSeq[6]. *, also identified when proportion inputs used for MaAsLin2, LinDA and ZicoSeq (Table S20).	

**Table S35:** Significantly differentially abundant pathways between sample types detected by ≥3 DA analysis tools at the late timepoint when counts are used as input for all tools.	
**Rinse samples**	**Swab Samples**
**Identified by 5 tools**	
GLUCONEO-PWY[12345*]<br>PYRIDNUCSYN-PWY[12345*]<br>PWY-7200[13456*]	
**Identified by 4 tools**	
HEMESYN2-PWY[1345*]<br>PWY-7953[1345*]<br>RIBOSYN2-PWY[1245*]	PWY-5676[1235*]
**Identified by 3 tools**	
1CMET2-PWY[134*]<br>PWY-5973[124*]<br>GLYCOLYSIS[124*]<br>PEPTIDOGLYCANSYN-PWY[124*]<br>PWY-6387[124*]<br>PWY-6700[124*]<br>PWY66-429[124*]<br>ARO-PWY[246]<br>PWY-6163[246]<br>PWY-7663[246]<br>PPGPPMET-PWY[145*]<br>PWY-5484[124*]<br>PWY-241[146*]<br>COLANSYN-PWY[146*]	VALSYN-PWY[356*]<br>P161-PWY[135]<br>PWY-6292[135]<br>PWY-6588[135]<br>PWY-6901[125]<br>PWY-7434[125]<br>UDPNACETYLGALSYN-PWY[125]<br>PWY-5981[156]
P-values are included in Table S7. Superscript represents tool: MaAsLin2[1], ANCOM-BC2[2], ALDEx2[3], DESeq2[4], LinDA[5] and ZicoSeq[6]. *, also identified when proportion inputs used for MaAsLin2, LinDA and ZicoSeq (Table S21).	
